# Untargeted Metabolomic Analysis of Sjögren–Larsson Syndrome Reveals a Distinctive Pattern of Multiple Disrupted Biochemical Pathways

**DOI:** 10.3390/metabo13060682

**Published:** 2023-05-23

**Authors:** Hongying Daisy Dai, Fang Qiu, Kimberly Jackson, Marcus Fruttiger, William B. Rizzo

**Affiliations:** 1 Department of Biostatistics, University of Nebraska Medical Center, Omaha, NE 68198, USA; daisy.dai@unmc.edu (H.D.D.); fqiu@unmc.edu (F.Q.); 2 Metabolon Inc., Morrisville, NC 27560, USA; kjackson@metabolon.com; 3 UCL Institute of Ophthalmology, University College London, London EC1V 9EL, UK; m.fruttiger@ucl.ac.uk; 4 Department of Pediatrics and Child Health Research Center, University of Nebraska Medical Center, Omaha, NE 68198, USA; 5 Children’s Hospital & Medical Center, Omaha, NE 68114, USA

**Keywords:** ichthyosis, spasticity, intellectual disability, fatty aldehyde, fatty alcohol, lipid metabolism, pathogenesis

## Abstract

Sjögren–Larsson syndrome (SLS) is a rare inherited neurocutaneous disease characterized by ichthyosis, spastic diplegia or tetraplegia, intellectual disability and a distinctive retinopathy. SLS is caused by bi-allelic mutations in *ALDH3A2*, which codes for fatty aldehyde dehydrogenase (FALDH) and results in abnormal lipid metabolism. The biochemical abnormalities in SLS are not completely known, and the pathogenic mechanisms leading to symptoms are still unclear. To search for pathways that are perturbed in SLS, we performed untargeted metabolomic screening in 20 SLS subjects along with age- and sex-matched controls. Of 823 identified metabolites in plasma, 121 (14.7%) quantitatively differed in the overall SLS cohort from controls; 77 metabolites were decreased and 44 increased. Pathway analysis pointed to disrupted metabolism of sphingolipids, sterols, bile acids, glycogen, purines and certain amino acids such as tryptophan, aspartate and phenylalanine. Random forest analysis identified a unique metabolomic profile that had a predictive accuracy of 100% for discriminating SLS from controls. These results provide new insight into the abnormal biochemical pathways that likely contribute to disease in SLS and may constitute a biomarker panel for diagnosis and future therapeutic studies.

## 1. Introduction

Sjögren–Larsson syndrome (SLS) is a rare inherited neurocutaneous disease characterized by ichthyosis, spastic diplegia or tetraplegia, intellectual disability and a distinctive retinopathy [1]. The skin disease is usually evident at birth, and neurologic symptoms appear by 1–2 years of age with the onset of spasticity and delay in achieving motor milestones. SLS is caused by bi-allelic inactivating mutations in *ALDH3A2*, which codes for fatty aldehyde dehydrogenase (FALDH) and results in abnormal lipid metabolism [2,3]. Although more than 100 mutations in *ALDH3A2* have been reported in SLS patients, no useful genotype–phenotype correlations have emerged [4,5]. The disease has a worldwide prevalence of approximately 1 in 340,000. 

Despite knowing the gene defect and enzyme abnormality, the pathogenic mechanisms in SLS are still unclear [6]. FALDH has a central role in metabolizing long-chain fatty aldehydes, which are generated from degradation of several diverse lipids. Fatty aldehydes are highly reactive molecules that are able to form covalent adducts with lipids and proteins [7], thereby potentially affecting unrelated cellular pathways. FALDH also acts as a component of fatty alcohol:NAD oxidoreductase (FAO) that catalyzes the oxidation of long-chain alcohols. A deficiency in FAO results in the accumulation of fatty alcohols [8], which are diverted into excessive synthesis of ether lipids in cultured keratinocytes [9], as well as the skin [10] and brain [11]. Consequently, lipid abnormalities in SLS may have secondary disruptive effects far removed from the primary biochemical defect, and could impact a variety of metabolic pathways that contribute to the development of symptoms. 

Investigations of inborn errors of metabolism have largely focused on the specific biochemical pathway altered, and effects beyond the primary pathway are generally unknown. Untargeted metabolomic studies, however, provide a powerful unbiased approach for identifying perturbed biochemical pathways [12,13] and show promise for the diagnosis of rare diseases [14,15,16,17,18,19,20,21,22,23]. 

To search for metabolic pathways disrupted in SLS, we performed untargeted metabolomic analysis of a group of 20 SLS individuals. These results reveal a unique metabolomic profile that points to previously unsuspected areas of metabolism that may contribute to SLS symptoms, and also identifies new potential biomarkers for future therapeutic studies.

## 2. Materials and Methods

### 2.1. Human Subjects

This research was approved by the Institutional Review Board at the University of Nebraska Medical Center. Twenty SLS subjects were enrolled in the Sterol and Isoprenoid Research Consortium of the Rare Diseases Clinical Research Network (Clinicaltrials.gov; NCT01971957; accessed on 30 October 2013). All SLS subjects were confirmed to carry mutations in *ALDH3A2*. An equal number of age- and sex-matched control subjects were also studied.

### 2.2. Procedures

#### 2.2.1. Preparation of Plasma

Fasting blood specimens were collected in EDTA-treated blood tubes and centrifuged at 3600× *g* for 10 min. Plasma aliquots were stored at −70 °C prior to analysis. 

#### 2.2.2. Metabolomic Analysis

Sample processing and untargeted mass spectrometry analysis were carried out at Metabolon Inc. (Morrisville, NC, USA) as previously described [13]. Briefly, samples were analyzed using ultra-high-performance liquid chromatography–tandem mass spectrometry. The biochemical peaks were compared to a reference library to identify metabolites based on retention time, molecular weight (*m*/*z*), ion spectra, preferred adducts, and in-source fragments. Metabolites were annotated and biochemical pathways mapped [24]. Peak area data were batch-normalized and imputed for missing values, using cube root transformation and Pareto scaling [25]. Normalized data were visually displayed in box plots as Scaled Intensity.

### 2.3. Statistical Analysis

The matched-pairs t-test was used to determine the statistical significance (*p* value) of metabolite mean differences between comparator groups (SLS and controls). False discovery rates, estimated using q values, were used to control for type I errors. In our analyses, the significantly upregulated or downregulated metabolites were defined based on three selections including (1) *p* < 0.05 and *q* < 0.05 [to control for false discovery rate]; (2) metabolite detected in 85% or more samples (% filled value ≥85%); and (3) fold ratio between SLS and Control >1.1 or <0.9. Our selection criteria were based on statistical and biological reasoning by following a previous metabolomic study using similar experimental measures and study design [26]. We chose a low threshold for fold change since the metabolites are relatively less variable as compared to gene expressions. Using a lower threshold also allowed us to capture affected pathways that might have been missed due to dilution of tissue-derived metabolites into plasma. We further performed random forest analyses—a decision-tree-based non-parametric machine learning method for supervised classification of disease status (SLS vs. control) using metabolites as predictors. Parametric statistical analysis was performed using SAS 9.4 (Cary, NC, USA), and random forest analysis was performed using R program (http://cran.r-project.org/; accessed on 30 November 2021).

## 3. Results

Twenty SLS subjects (13.0 ± 7.3 years old, range 4–30 years) consisting of 9 males and 11 females were investigated. An equal number of age- and sex-matched control subjects (13.0 ± 7.5 years old) were concurrently studied. 

In plasma, 1041 biochemicals were detected, including 823 identified and 218 unknown ones. We focused on identified biochemicals only and classified mean values as significantly different between SLS and controls if they fulfilled the following criteria: (1) *p* ≤ 0.05 and *q* ≤ 0.05; (2) filled values ≥85%; and (3) SLS/control fold ratio >1.1 or <0.9. Of the identified biochemicals, 121 (14.7%) were found to quantitatively differ in the overall SLS cohort compared to controls (Table 1). These are referred to as “significant metabolites”. Seventy-seven of the significant metabolites were decreased in SLS and 44 were increased.

### 3.1. Stratified Analysis by Sex

When biochemical levels were stratified by sex, we found 37 metabolites that differed significantly between SLS males vs. male controls, and 77 metabolites that differed by disease status in females. In contrast, there were no significant metabolites that differed by gender within SLS subjects and within control subjects, respectively (see Table 1). 

A detailed list of significant metabolites identified between SLS and controls for the overall analysis and stratified analysis by gender can be found in Supplementary Tables S1–S4. 

### 3.2. Metabolic Super Pathways 

The significant metabolites in SLS can be classified into several large metabolic super pathway categories. For an overview of the disrupted metabolic pathways in SLS, we examined the number and percentage of significant metabolites seen in each of the super pathways (Table 1). Two super pathways, Amino Acids and Lipids, had the largest numbers of significant metabolites associated with disease status, with 43 out of 210 (20.5%, *p* = 0.0285) significant metabolites in the Amino Acid super pathway and 41 of 292 (14.0%, *p* = 0.6415) significant metabolites in the Lipids super pathway. Among relatively small-sized super pathways, the Carbohydrate super pathway (8 out of 23, 34.8%, *p* = 0.0154) and the Cofactors and Vitamins super pathway (10 out of 37, 27.0%, *p* = 0.0422) also had high percentages of significant metabolites. Similar patterns were identified in the stratified subpathway analyses (Supplementary Table S1). The number of significant metabolites were doubled among females than among males for super pathways involving amino acids, cofactors and vitamins, lipids, nucleotides, and xenobiotics. 

### 3.3. Metabolic Subpathways

Significant metabolites in super pathways were further classified according to their subpathways (Table 2 and Supplementary Tables S1–S4). Among a total of 100 subpathways for all 823 metabolites, we identified 52 subpathways with 121 differentially expressed biochemicals. We compared the difference between SLS and control for the overall sample (males and females). Within the Amino Acid super pathway, 15 subpathways were identified comprising 210 identified metabolites. Significant differences (n = 43) involving at least one metabolite were seen in 14 of these subpathways. The subpathway “Methionine, Cysteine, S-Adenosylmethionine (SAM) and Taurine Metabolism” had seven (32, *p* = 0.0366) significant metabolites, with five metabolites having >1.1-fold change and two metabolites having <0.9-fold change between SLS vs. control (Table 2 and Supplementary Table S1). The “Tryptophan Metabolism” subpathway had seven (30%, *p* = 0.0459) significant metabolites, with six metabolites having <0.9-fold change between SLS vs. control. Similarly, the “Phenylalanine Metabolism” subpathway had four significant metabolites (57%, *p* = 0.0119), all decreased in SLS. Other amino acid subpathways had 1–5 significant metabolites. 

Within the Carbohydrate super pathway (n = 23 metabolites), the “Glycolysis, Gluconeogenesis, and Pyruvate Metabolism” subpathway had four of six significant metabolites (67%, *p* = 0.0058), with three metabolites having >1.1-fold change and one metabolite having <0.9-fold change between SLS vs. controls. The “Glycogen Metabolism” subpathway had 2 (100%, *p* = 0.0221) significant metabolites, all with >1.1-fold change between SLS vs. controls.

Within the Cofactors and Vitamins super pathway, four of seven (57%, *p* = 0.0119) metabolites in the “Vitamin A Metabolism” subpathway were decreased in SLS as compared to controls. In the “Nicotinate and Nicotinamide Metabolism” subpathway, three of eight metabolites (38%, *p* = 0.1029) were significantly different in SLS.

The Lipids super pathway comprised 42 subpathways, each ranging from 1–33 identified metabolites. Ten of these subpathways had at least two significant metabolites that differed in SLS (Table 2). Of six medium chain acylcarnitine metabolites measured in the “Fatty Acid Metabolism, Acylcarnitine, Medium Chain” subpathway, four were significantly decreased and none increased. Similarly, only decreases (2 of 10 measured) were seen in “Fatty Acid, Monounsaturated Acylcarnitine” subpathway and “Fatty Acid Dicarboxylate” subpathway (2 of 33 measured). In contrast, metabolites in the “Phospholipid Metabolism” subpathway had four of six (67%, *p* = 0.0058) significant metabolites, all with > 1.1-fold difference between SLS vs. control. The ”Sterol Metabolism” subpathway (4 of 8 measured; 50%, *p* = 0.021), “Primary Bile Acid Metabolism” (3 of 11; 27%, *p* = 0.2163) and “Secondary Bile Acid Metabolism” (7 of 20; 35%, *p* = 0.0219) subpathways were all decreased, while the significant metabolites in the ”Phosphatidylserine” (2 out of 2 measured; 100%, *p* = 0.0221), “Sphingolipid Synthesis” (3 out of 4 measured; 75%, *p* = 0.0118), and “Sphingosines” (2 out of 2 measured; 100%, *p* = 0.0221) were all increased. 

Twenty-nine subpathways had at least two metabolites that differed between SLS and controls (Table 2). In 8 subpathways, all measured metabolites were increased in SLS and in 13 subpathways, all metabolites were decreased. Eight subpathways had some metabolites that were increased and others decreased.

**Table 2:** Metabolic subpathways with at least two significant metabolites that differ in SLS and controls. Data listed for all SLS subjects combined (male and female). See Supplementary Table S1 for a more extensive summary of all subpathways.

Super Pathway	Subpathway	Metabolites Measured	SLS/Control > 1.1	SLS/Control < 0.9
Amino Acid	Arginine and Proline Metabolism	21	0	5
	Glutamate Metabolism	12	2	0
	Glutathione Metabolism	7	1	1
	Histidine Metabolism	16	0	4
	Leucine, Isoleucine and Valine Metabolism	31	0	2
	Lysine Metabolism	20	2	1
	Methionine, Cysteine, SAM and Taurine Metabolism	22	5	2
	Phenylalanine Metabolism	7	0	4
	Tryptophan Metabolism	23	1	6
	Tyrosine Metabolism	19	0	3
Carbohydrate	Glycogen Metabolism	2	2	0
	Glycolysis, Gluconeogenesis, and Pyruvate	6	3	1
Cofactors and Vitamins	Nicotinate and Nicotinamide	8	1	2
	Vitamin A	7	0	4
Lipid	Fatty Acid (Acylcarnitine, Medium Chain)	6	0	4
	Fatty Acid (Acylcarnitine, Monounsaturated)	10	0	2
	Fatty Acid, Dicarboxylate	33	0	2
	Phospholipid Metabolism	6	4	0
	Phosphatidylserine Metabolism	2	2	0
	Primary Bile Acid Metabolism	11	0	3
	Secondary Bile Acid Metabolism	20	0	7
	Sphingolipid Synthesis	4	3	0
	Sphingosines	2	2	0
	Sterol Metabolism	8	0	4
Nucleotide	Purine Metabolism, (Hypo)Xanthine/Inosine containing	8	2	1
	Purine Metabolism, Adenine containing	7	2	0
Partially Characterized Molecules	Partially Characterized Molecules	24	0	2
Energy	TCA Cycle	9	2	0
Xenobiotics	Chemical	22	1	3

### 3.4. Random Forest Analysis

**Figure 1:**
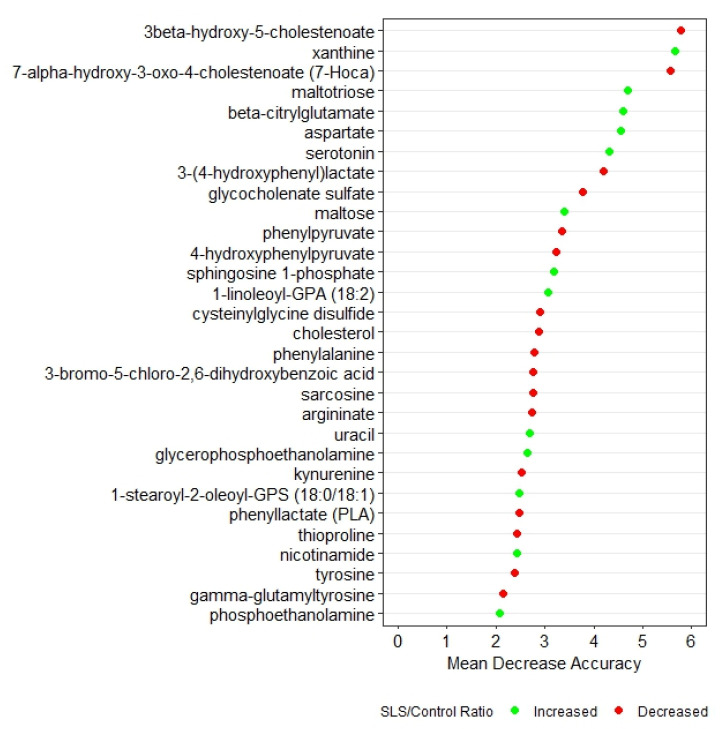
Random forest analysis of the top 30 metabolites that distinguish SLS patients from controls. Green symbols indicate metabolites that were increased in SLS, whereas red symbols indicate decreased metabolites.

Random forest analysis based on q-values identified the top 30 biochemicals that differed between SLS and controls (Figure 1). These biochemicals represented 7 super pathways and 20 subpathways and had a predictive accuracy of 100%, indicating very distinct profiles. Box plots of each of the 30 metabolites in SLS and controls are compared in Supplementary Figure S1. 

### 3.5. Metabolite Interrogation

Pathway analyses of the 121 significant metabolites identified at least seven major areas impacted in SLS that involve metabolism of sphingolipids, sterols, bile acids, glycogen, purines and certain amino acids. Supplementary Table S1 lists the number of significant metabolites in the subpathways for the overall SLS and control groups. Individual metabolites identified are listed in Table S2, while Tables S3 and S4 provide further comparisons, stratified by gender. To illustrate the extremes in significant metabolite variation, the top 15 metabolites that are increased or decreased in SLS are shown in Supplementary Figure S2. We point out some of the most noteworthy differences. 

#### 3.5.1. Sphingolipid Metabolism

**Figure 2:**
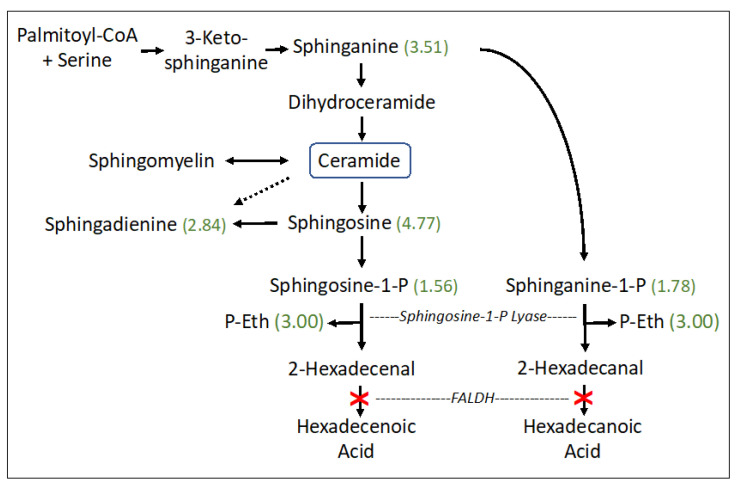
Sphingosine and ceramide pathway in SLS. Six metabolites were measured. Statistically significant metabolites in SLS are indicated by a number in parentheses corresponding to the ratio of mean SLS to mean controls. Metabolites that lack an associated number were either not measured or did not differ between the groups. Dashed arrow indicates a minor contribution of ceramide to sphingodienine synthesis. Deficiency of FALDH in SLS is indicated by a red X. FALDH, fatty aldehyde dehydrogenase; P-Eth, phosphoethanolamine.

The sphingolipid pathway appeared abnormal (*p* = 0.0118) in SLS with striking accumulations of sphingosine (4.77-fold), sphinganine (dihydrosphingosine) (3.51-fold), sphingadienine (2.84-fold), and lesser increases in phosphorylated sphingosines: sphingosine-1-phosphate (S1P) (1.56-fold) and sphinganine-1-phosphate (dihydrosphingosine-1-phosphate; dhS1P) (1.78-fold). S1P and dhS1P degradation, catalyzed by S1P lyase, generates fatty aldehydes 2-hexadecenal (from S1P) or hexadecanal (from dhS1P), respectively, and phosphoethanolamine (P-Eth) as a coproduct (Figure 2). SLS subjects are deficient in FALDH, which is responsible for oxidizing 2-hexadecenal to fatty acid [27]. The P-Eth product of S1P lyase was also found to be elevated 3.00-fold, although it is also produced during other metabolic reactions. 

#### 3.5.2. Sterol and Bile Acid Metabolism

**Figure 3:**
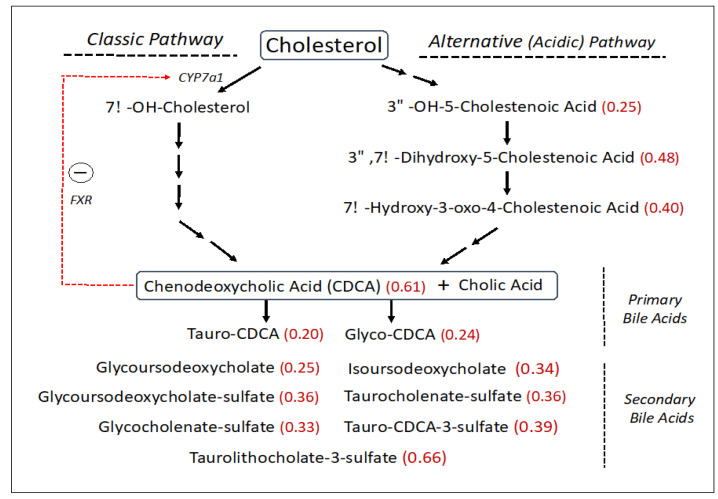
Bile acid synthesis pathway (abbreviated) in SLS. Thirty-one metabolites were measured. Statistically significant metabolites in SLS are indicated by a number in parentheses corresponding to the ratio of mean SLS to mean control. Metabolites that lack an associated number were either not measured or did not differ between the groups. Red arrow indicates a feedback effect of bile acids on *CYP7a1* gene expression. FXR, farnesoid X-receptor. CDCA, Chenodeoxycholic acid.

SLS subjects had reduced levels of free cholesterol (0.76-fold), which is a central precursor for other sterols including bile acids. The synthesis of bile acids accounts for about 50% of the daily serum cholesterol turnover. Bile acids are made via two biosynthetic pathways: the classic (or neutral) pathway, which accounts for the major flux of cholesterol in the liver, and the minor alternative (or acidic) pathway with some reactions carried out in other non-hepatic tissues (Figure 3). The SLS cohort demonstrated a decrease in several cholesterol-derived metabolites associated with the alternative bile acid pathway, including 3ß-hydroxy-5-cholestenoate (0.25-fold), 7α-hydroxy-3-oxo-4-cholestenoate (7-Hoca) (0.40-fold) and 3ß,7α-dihydroxy-5-cholestenoate (0.48-fold). The liver produces two major primary bile acids: cholic acid and chenodeoxycholic acid (CDCA), which undergo further modifications. In the SLS cohort, there were significant reductions in CDCA (0.61-fold), glyco-CDCA (0.24-fold), and tauro-CDCA (0.20-fold) compared to the control population, while cholic acid was non-significantly altered. Seven secondary bile acids including five sulfated bile acids were also reduced (0.25–0.66-fold) in SLS (Figure 3). Unlike most other metabolomic changes, however, the significant reductions in primary and secondary bile acids in SLS as a group were largely driven by the female population, although males showed similar trends (compare Supplementary Tables S3 and S4). 

#### 3.5.3. Carbohydrate Metabolism

Two degradation metabolites of glycogen were markedly elevated in the SLS group. SLS subjects had higher levels of maltose (8.8-fold) and maltotriose (13.27-fold), consistent with increased breakdown of glycogen. Both of these metabolites were among the top 10 discriminating biochemicals in our random forest analysis (Figure 1). 

#### 3.5.4. Purine Metabolism

Four of five metabolites in the purine metabolism subpathway were increased in SLS. Adenosine-5′-monophosphate (AMP), hypoxanthine, xanthine and adenine were increased by 4.02-fold, 1.42-fold, 1.94-fold and 2.1-fold, respectively, suggesting a general effect on purine metabolism. Adenosine triphosphate (ATP) and inosine triphosphate (ITP) levels were not measured. 

#### 3.5.5. Vitamin and Cofactor Metabolism

There were significant reductions in four of seven measured vitamin A metabolites with mean levels at 0.54–0.71-fold in the SLS subjects. One vitamin B6 metabolite (pyridoxal) also decreased (0.82-fold). Alpha-tocopherol was reduced (0.74-fold), whereas ß/∂ tocopherols were not significantly changed. In contrast, nicotinamide was elevated (2.86-fold).

#### 3.5.6. Amino Acid Metabolism

**Figure 4:**
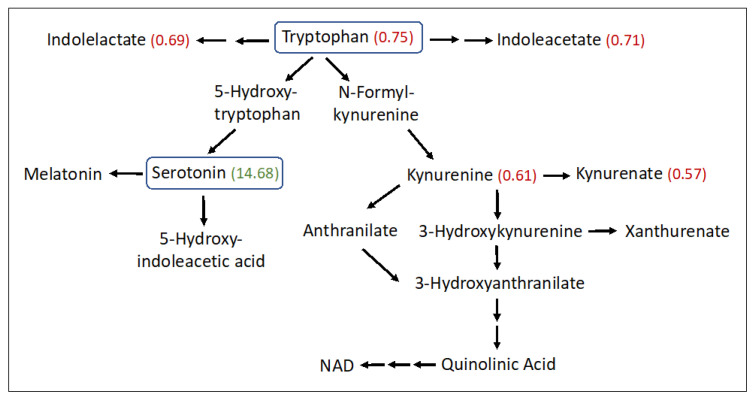
Tryptophan metabolism pathway in SLS. Twenty-three metabolites were measured. Statistically significant metabolites in SLS are indicated by a number in parentheses corresponding to the ratio of the mean SLS to the mean control. Metabolites that lack an associated number were either not measured or did not differ between the groups. Note that oxindolylalanine (SLS/control = 0.58, Supplementary Table S2), which is a product of bacterial tryptophan metabolism in the intestine, is not shown.

Significant abnormalities were seen in 14 amino acid subpathways (Tables S1 and S2), and 10 of these had two or more significant metabolites detected (Table 2). Several amino acids were found to be increased in the SLS group, particularly aspartate (1.9-fold), glutamate (1.86-fold), beta-citrylglutamate (2.99-fold), 2-aminoadipate (1.53-fold), 5-oxoproline (1.2-fold), S-adenosylhomocysteine (1.62-fold), taurine (1.96-fold), hypotaurine (2.37-fold) and N-acetyltaurine (1.54-fold). In contrast, several other amino acids were decreased in SLS. For example, phenylalanine (0.76-fold) and three of its metabolites were reduced (Table 2 and Table S2). Tryptophan (0.75-fold) and several of its metabolites (kynurenine, kynurenate, indoleacetate and oxindolylalanine) were decreased in SLS subjects to 57–75% of the mean control levels (Figure 4). In contrast, serotonin (5-hydroxytryptamine) showed a striking 14.68-fold mean elevation. Cysteine (0.81-fold) and cystine (0.61-fold) were also reduced in SLS but cysteine-S-sulfate was notably increased (3.85-fold). Arginine (0.68-fold) along with several other amino acids and/or their metabolites were either mild-moderately low or not significantly altered. 

## 4. Discussion

We identified a distinct metabolomic profile in SLS plasma that points to secondary alterations in several biochemical pathways that were heretofore not suspected. It is possible that these pathways are impacted directly via the formation of fatty aldehyde adducts with key proteins or indirectly through fatty alcohol-dependent lipid changes in cellular membranes. In either case, the metabolite alterations in SLS can arise from the modulation of enzymes, transporters, regulatory proteins or signaling lipids that impact these pathways. Consequently, plasma metabolite concentrations do not solely reflect the metabolic activity in tissues but are influenced by complex interactions involving metabolite transfer into and out of blood and contributions from many tissues. Furthermore, the relative changes in blood metabolites point to biochemical pathways that may be perturbed to a greater or lesser extent in specific tissues. Although we found several biochemical pathways to be disrupted in SLS, it is likely that additional pathways are also affected, which are better revealed by more targeted biochemical analyses such as lipidomics. The potential clinical impact of the abnormalities detected in SLS is likely to depend on the particular biochemical pathway involved, the degree of metabolite abnormality, and the physiological role of each metabolite within the pathway. Consequently, it should not be assumed that all of the disrupted pathways revealed through our metabolomic analyses have clinical importance in SLS. Although the underlying biochemical defect in SLS was discovered using cultured cell models [1,3,6], our results underscore the global and complex effects of FALDH deficiency that can only be appreciated at an organismic level.

Sphingolipid metabolism is the only abnormal pathway detected in our metabolomic study that is known to be directly connected to defective fatty aldehyde metabolism in SLS. It is a highly regulated pathway that controls the synthesis and degradation of ceramide and S1P, which are two bioactive lipids that are intimately involved in many cellular processes [28,29]. Sphinganine (dihydrosphingosine) is a biosynthetic precursor to ceramide and sphingosine is a catabolic product of ceramide degradation (Figure 2). Sphingosine and sphinganine are phosphorylated by sphingosine kinase to produce S1P and sphinganine-1-phosphate (dhS1P), respectively, which in turn are catabolized by S1P lyase to generate 16-carbon fatty aldehyde products (e.g., 2-hexadecenal from S1P and hexadecanal from dhS1P) and P-Eth. 2-Hexadecenal is normally oxidized to fatty acid by FALDH, which is deficient in SLS, and subsequently hydrogenated to palmitic acid and eliminated by mitochondrial ß-oxidation or incorporated into cellular lipids [27]. The S1P lyase reaction is not known to be inhibited by its fatty aldehyde or P-Eth products [30]. The accumulation of S1P, dhS1P, sphingosine, sphinganine and sphingadienine together with P-Eth in SLS plasma suggests that the sphingolipid pathway is upregulated, perhaps leading to increased production of 2-hexadecenal, which cannot be oxidized by FALDH. This fatty aldehyde can induce apoptosis in cultured cells via a JNK-mediated pathway [31] and covalently binds to Bax, a pro-apoptotic protein [32]. It may be speculated that increased apoptosis contributes to certain pathologic findings in SLS such as the thinning of the retinal layers [33] or myelin deficiency [6,11]. However, lipidomic analysis of one autopsied SLS brain did not detect abnormalities in sphingosine, S1P or ceramides [11], suggesting that the S1P pathway in SLS may be selectively disrupted in other extra neural tissues. Interestingly, the ceramide concentration in SLS plasma, measured by lipidomic analysis, was mildly decreased to 0.82-fold. 

The accumulation of S1P (and dhS1P) in SLS may have important biological effects [29]. S1P is a key bioactive molecule that is involved in regulating a variety of pathways such as cell growth, differentiation, inflammation and apoptosis. It functions as an intracellular signaling molecule and also exerts extracellular effects by interacting with five distinct S1P receptors on cell plasma membranes [34]. In blood, S1P levels reflect a complex contribution of synthesis in various tissues and release from blood cells, platelets and vascular endothelial cells [35]. Changes in blood S1P have been reported in a number of different disease states including cancer, inflammatory disorders [36], diabetes [37], obesity [38], liver disease [39], and neurodegenerative diseases [40], thereby implying a key role in many tissues. 

In the skin, synthesis and metabolism of sphingolipids including ceramide, glucosylceramide, omega-hydroxy-(acyl)-ceramide and S1P are critical lipids for forming and maintaining the epidermal water barrier [41,42,43]. S1P inhibits keratinocyte proliferation, stimulates calcium influx and induces cell differentiation genes [44]. As keratinocytes undergo differentiation at the stratum granulosum–stratum corneum interface, they synthesize and release precursor membranes before undergoing apoptosis and forming dead corneocytes in the stratum corneum (SC). The extracellular SC membranes self-assemble into multilamellar membranes that are covalently attached to the corneocytes. The membranes are rich in ceramides along with cholesterol and free fatty acids, and functionally constitute the water barrier. In SLS, these membranes are deficient in certain acylceramides [45,46,47] and accumulate ether lipids [10], which disrupts the water barrier [48]. A leaky water barrier is a common pathologic feature of most ichthyoses [49]. The abnormal sphingolipid profile in SLS plasma, if reflected in the skin, is expected to profoundly impact epidermal differentiation and would likely contribute to the mechanisms, leading to ichthyosis. Indeed, genetic deficiency of S1P lyase results in the accumulation of S1P, sphingosine and ceramide and is associated with ichthyosis along with other multisystem symptoms [50]. 

Alterations in sterol metabolism, including a 24% reduction in free cholesterol, were unexpectedly seen in the SLS cohort. Cholesterol esters constitute the major form of this sterol in serum [51]. Although not measured in our metabolomic study, cholesterol esters were comparably reduced in a companion lipidomic study (manuscript in preparation). Cholesterol is a key lipid in membranes throughout the body and is a central precursor for synthesis of steroid hormones in the adrenal glands, ovaries and testes, along with neurosteroids in the brain. 

Cholesterol incorporation into the bile acid biosynthetic pathway in the liver accounts for a major metabolic flux. Bile acid synthesis occurs through a complex series of reactions via two somewhat different pathways that converge into the production of cholic acid and chenodeoxycholic acid (CDCA) [52]. The classic (neutral) biosynthetic pathway is the major source of bile acid production [53], whereas the alternative (acidic) pathway contributes quantitatively less to the total biosynthesis of bile acids in humans [54]. In SLS, significant reductions to 25–40% of the mean control were detected in several metabolites comprising the alternative bile acid pathway, particularly 3ß-hydroxy-5-cholestenoate, 7α-hydroxy-3-oxo-4-cholestenoate and 3β,7α-dihydroxy-5-cholestenoate (Figure 3). Cholic acid and CDCA may be conjugated with glycine and taurine, providing further heterogeneity beyond the primary bile acids. In the overall SLS cohort, CDCA was decreased significantly (61% of mean control) along with glyco-CDCA (24% of control) and tauro-CDCA (20% of control). Cholic acid and its metabolites tended to be less affected than CDCA. 

Bile acids are normally synthesized in the liver, stored in the gall bladder and secreted into the intestine to aid fat digestion [52,53]. The biochemical transformation of primary bile acids into secondary bile acids occurs in the gut by the action of bacteria. The primary and secondary bile acids are subsequently reabsorbed into the blood and transported back to the liver via the enterohepatic circulation. The sulfation of bile acids decreases their intestinal absorption and increases their urinary and fecal excretion [55]. Consequently, blood levels of bile acids in SLS can be impacted at any step in this process. Seven secondary bile acids detected in the overall SLS cohort were reduced to 25–66% of the mean control, and five of these were sulfated. The bile acid reductions in the overall SLS cohort were largely accounted for by the female subjects who had even greater reductions compared to SLS males (compare Supplementary Tables S3 and S4). Gender differences in blood bile acid concentrations in the normal population are known to exist [56]. Females have lower levels of certain bile acids along with the total bile acid concentration, perhaps making SLS females more susceptible to disruption in the bile acid pathways. Reductions in secondary bile acid metabolites in SLS might be attributable in part to differences in primary bile acids available in the gut, altered enterohepatic circulation, or changes in the intestinal microbiome. If the decrease in serum bile acids is reflected in the intestine, the reduced serum cholesterol levels seen in SLS may arise in part from impaired dietary absorption. 

Reduced bile acids in SLS may have significance beyond their well-known role in fat digestion. Bile acids and their metabolites act as signaling molecules through their interaction with several nuclear receptors including the farnesoid X receptor (FXR), pregnane X receptor (PXR) and vitamin D receptor (VDR), and by activation of membrane-bound receptors including sphingosine-1-phosphate receptor 2 (S1PR2) and Takeda G protein receptor 5 (TGR5) [57]. CDCA is the most potent activator of FXR, which interacts with FGF15/19 and activates JNK1/2 and ERK1/2 signaling pathways to decrease expression of *CYP7A1*, which encodes the rate-limiting enzyme in the classic bile acid synthesis pathway [58]. In such a manner, bile acid synthesis is regulated through feedback inhibition. With reduction of CDCA in SLS, *CYP7A1* expression is expected to increase and lead to enhanced bile acid synthesis through the classic pathway, perhaps shunting precursors away from the acidic pathway and accounting for the reduction of several acidic pathway intermediates in SLS. 

A reduction in bile acids in SLS may impact multiple biochemical pathways. Bile acid signaling via FXR and S1PR2 has been implicated in gene expression in the central nervous system and involvement in several neurodegenerative diseases [59]. S1PR2 is also important in liver carbohydrate and lipid metabolism [60]. The activation of S1PR2 by conjugated bile acids stimulates the downstream ERK1/2 and AKT signaling pathways, which lead to the downregulation of glycogen synthase and gluconeogenesis genes [61,62]. Bile acid activation of the ubiquitous membrane-associated TGR5 receptor increases cAMP and activates cAMP response element binding protein, thereby inducing production of thyroxine and increasing energy expenditure [63]. Both 3β-hydroxy-5-cholestenoate and 3β,7α-dihydroxy-5-cholestenoate, which are decreased in SLS plasma, are liver-X receptor (LXR) ligands that can influence motor neuron survival in vitro [64,65]. Each of these pathways are complex and likely modulated by mechanisms beyond bile acids, so whether the bile acid reductions in SLS plasma are sufficient to affect these pathways remains to be determined.

Serotonin (5-hydroxytrypamine, 5-HT) is a product of tryptophan metabolism. The initial synthesis of 5-hydroxytryptophan, catalyzed by tryptophan hydroxylase-1 (Tph1) in peripheral tissues, is followed by decarboxylation to 5-HT (Figure 4). The striking elevation of plasma serotonin and reduction of other tryptophan metabolites in SLS plasma suggests preferential shunting of tryptophan towards serotonin synthesis. Serotonin has been reported to be elevated in the autopsied brain of one SLS patient along with a reduction in dopamine [66]. Although serotonin is a well-known neurotransmitter in the brain, blood levels of the monoamine are largely derived from the intestinal enterochromaffin (EC) cells. However, peripheral serotonin does not cross into the brain and therefore elevated plasma serotonin in SLS should not directly impact the CNS-related symptoms.

The elevated plasma serotonin in SLS may arise from numerous mechanisms including enhanced synthesis, impaired breakdown, or proliferation of EC cells in the intestine. Serotonin has multiple effects outside the brain including the regulation of glucose homeostasis, lipid metabolism, obesity and potential effects on type 2 diabetes [67]. EC cells are capable of sensing ingested nutrients and are therefore influenced by diet and the microbiome. Serotonin stimulates hormone sensitive lipase via activation of the 5-HT2b receptor and raises plasma free fatty acids and glycerol [68]. It regulates plasma glucose in part by binding to 5-HT2b receptors on liver hepatocytes and modulating activity of rate-limiting enzymes glucose-6-phosphatase and fructose 1,6-bisphosphatase via transcriptional enhancement [68]. Serotonin also influences hepatic glucose metabolism through interaction with a repertoire of 5-HT receptors, which in part regulate blood glucose transport, glycogen phosphorylase activity and glycogen synthesis [69]. In the SLS subjects, we found highly elevated maltose and maltotriose, two metabolites of glycogen breakdown that suggested dysregulated glycogen metabolism, perhaps related to serotonin elevations. 

In addition to its metabolic effects, serotonin is implicated in sensory itch, a common clinical feature of SLS. Serotonin is one of several molecules that induce itch. Serotonin injected into the skin of mice evokes itch by activating 5-tryptamine receptor(s) (5-HTR_2_, 5-HT_7_) on primary sensory afferent neurons, which in turn activates TRPA1 and TRPV4 ion channels, leading to calcium influx and neuron firing [70,71]. Itch in patients with chronic pruritus is decreased by treatment with selective serotonin reuptake inhibitors (SSRI) such as fluvoxamine and paroxetine [72,73]. However, serum serotonin levels do not correlate highly with the clinical severity of itch in children with chronic cholestatic liver disease [74]. The mechanisms of itch may differ between diseases and it is unclear whether elevated serotonin might contribute to the pruritus in SLS.

Elevations in TCA cycle-related metabolites (succinate, citraconate/glutaconate, lactate, and pyruvate) suggest that energy demand may be impacted in SLS. Measurements of ATP, which were not done in this metabolomic study, would be necessary to support this hypothesis. More definitive answers will require studies in cultured SLS cells to look at mitochondrial function and energy production. In addition, elevations of AMP, adenine, xanthine and hypoxanthine suggest that the purine pathway is stimulated in SLS.

Amino acid metabolism comprises a large number of the subpathways found to be affected in SLS. Both increases and decreases in amino acids were seen, which may be significant beyond their involvement in protein synthesis. For example, elevations in aspartate and glutamate are metabolically related to Krebs cycle activity and are known excitatory neurotransmitters. If the elevations seen in plasma also occur in the brain of SLS patients, they may contribute to the neurologic symptoms. Taurine, a ß-amino acid that is not incorporated into protein, and two of its metabolites were also increased in SLS. Taurine is synthesized in the liver and derived from the diet. It has potent antioxidant activity and levels are greatly increased in tissues such as brain, retina, skeletal muscle and heart, where oxidative stress is prominent [75]. The increase in taurine is notable in the context of reduced tauro-conjugated bile acids, suggesting that levels of this amino acid do not drive bile acid conjugation.

Similar to vitamins, nine essential amino acids are derived solely from the diet. Two essential amino acids, phenylalanine and tryptophan, were reduced in the SLS cohort, which likely accounts for the commensurate decrease in some of their metabolic products. Although dietary protein intake was not strictly controlled in our study, all SLS subjects reported consuming a regular diet with no unusual protein restrictions. Furthermore, since most essential amino acids were not reduced in SLS, we conclude that dietary protein intake was probably normal. This suggests that reduced phenylalanine and tryptophan levels in SLS arise from selective impairment in their intestinal uptake or decreased renal tubular reabsorption.

Beyond revealing biochemical pathways that may be important in disease pathogenesis, the metabolomic profile seen in this study revealed new potential biomarkers for SLS. Currently, all easily accessible in vivo biomarkers for this disease are limited to targeted lipids related to FALDH deficiency such as elevated plasma fatty alcohols [8], urinary leukotriene B4 [76] and cutaneous ether lipids [10], or secondary reductions of certain cutaneous ceramides [45,46,47]. In contrast, the abnormal metabolomic profile in SLS constitutes a more global panel of potential biomarkers that may prove useful for diagnosis and monitoring the efficacy of future therapeutic trials [14,15,16,17,21]. In this regard, it will be important to determine the disease specificity of the SLS profile and its correlation with symptom severity.

There are several limitations and confounding factors that may have affected our study. First, SLS is an ultra-rare disease. Although we investigated the largest SLS cohort assembled in the United States, the number of subjects studied was still relatively small for statistical purposes. Second, we included a wide age range from children to adults, which probably added to metabolic variation and may have hidden age-dependent abnormalities. Third, some statistically significant, but modest, metabolite changes in SLS may be tolerated owing to the presence of counter-regulatory mechanisms that limit their clinical impact. It follows that those biochemical pathways with the greatest changes are more likely to have clinical effects. Fourth, metabolite changes in plasma may not faithfully reflect the presence or magnitude of metabolic changes within tissues that are pathologically affected in SLS, such as the brain, skin and retina. It is therefore important to confirm our findings in future studies that explore tissue-specific alterations in the biochemical pathways identified here.

In conclusion, the abnormal metabolomic profile detected in SLS plasma revealed in vivo secondary biochemical changes that may be critical for understanding the pathogenic mechanisms in this disease. Importantly, some of the disrupted biochemical pathways identified here are potentially amenable to therapeutic intervention. Finally, the distinct metabolomic profile in SLS identifies surrogate biomarkers that might prove useful for diagnosis and monitoring efficacy of future drug trials.

## Figures and Tables

**Table 1:** Metabolic super pathways affected in SLS. Metabolites were compared between SLS and control subjects, both overall and stratified by sex and metabolic pathways.

ANOVA Contrasts	SLS vs. Control				Male vs. Female	
Stratified Analysis	Overall		Male	Female	SLS	Control
Total Biochemicals (n = 823)	121 (77↓|44↑)		37 (16 ↓|21↑)	77 (40↓|37↑)	0	0
Super Pathway		*p*-value				
Amino Acid (n = 210)	43 (31|12)	0.0285	11 (7|4)	21 (12|9)	0	0
Carbohydrate (n = 23)	8 (1|7)	0.0154	4 (0|4)	7 (1|6)	0	0
Cofactors and Vitamins (n = 37)	10 (8|2)	0.0422	1 (0|1)	3 (2|1)	0	0
Energy (n = 10)	2 (0|2)	0.4478	1 (0|1)	1 (0|1)	0	0
Lipid (n = 292)	41 (27|14)	0.6415	15 (6|9)	34 (21|13)	0	0
Nucleotide (n = 38)	7 (2|5)	0.3306	3 (1|2)	6 (1|5)	0	0
Partially Characterized Molecules (n = 24)	2 (2|0)	0.8860	0 (0|0)	1 (1|0)	0	0
Peptide (n = 34)	2 (1|1)	0.9679	1 (1|0)	0 (0|0)	0	0
Xenobiotics (n = 155)	6 (5|1)	0.9999	1 (1|0)	4 (2|2)	0	0

These differentiating metabolites were selected based on three criteria including (1) *p* ≤ 0.05 and *q* ≤ 0.05; (2) % filled values ≥ 85%; and (3) fold ratio > 1.1 or <0.85. The total number of differentiating metabolites reported in each cell is followed by the number of significantly decreasing metabolites (↓) and the number of increasing metabolites (↑).

## Data Availability

Deidentified data supporting this research is available upon request to W.B.R. The raw data are not publicly available due to privacy.

## Supplementary Materials

The following supporting data can be downloaded at: https://www.mdpi.com/article/10.3390/metabo13060682/s1. Table S1: Subpathways with differential metabolite profiling between SLS and control, overall and stratified by sex. Table S2: Metabolites with differential profiling between SLS and control among all subjects (male and female) [77,78,79]. Table S3: Metabolites with differential profiling between SLS and control among females [77,78,79]. Table S4: Metabolites with differential profiling between SLS and control among males [77,78,79]. Figure S1: Boxplot comparisons of metabolites identified via Random Forest analysis. The y-axis in all box plots corresponds to Scaled Intensity of normalized raw data (see Procedures, Metabolomic Analysis), not SLS/Control ratios. Figure S2: Top 15 metabolites that were increased or decreased in SLS. A. Increased metabolites. B. Decreased metabolites. x-axis represents mean SLS/Control ratio.
