# Supplementary material for: Untargeted Metabolomic Analysis of Sjögren–Larsson Syndrome Reveals a Distinctive Pattern of Multiple Disrupted Biochemical Pathways
Source: Metabolites. 2023 May 23;13(6):682. doi: 10.3390/metabo13060682 (PMC10301067; doi:10.3390/metabo13060682)
Supplement: Supplementary file 1 [file metabolites-13-00682-s001.zip › metabolites-2334388-supplementary.pdf]

SUPPLEMENTARY MATERIALS

Supplementary Table S1. Subpathways with differential metabolic profiling between SLS and control, overall and stratified by sex.

		Metabolites			SLS vs. Control			Male SLS vs. Male Control			Female SLS vs. Female Control		
Super Pathway	Subpathway	Metabolites Measured	p-value	%	Significant	>1.1	<0.9	Significant	>1.1	<0.9	Significant	>1.1	<0.9
Amino Acid	Alanine and Aspartate Metabolism	8	0.7209	13%	1	1	0	1	1	0	1	1	0
	Creatine Metabolism	3	0.3813	33%	1	0	1	0	0	0	0	0	0
	Glutamate Metabolism	12	0.5474	17%	2	2	0	1	1	0	2	2	0
	Glutathione Metabolism	7	0.2782	29%	2	1	1	0	0	0	1	0	1
	Glycine, Serine and Threonine Metabolism	12	0.8518	8%	1	0	1	0	0	0	2	1	1
	Guanidino and Acetamido Metabolism	2	1	0%	0	0	0	0	0	0	0	0	0
	Histidine Metabolism	16	0.205	25%	4	0	4	1	0	1	1	0	1
	Leucine, Isoleucine and Valine Metabolism	31	0.9528	6%	2	0	2	0	0	0	0	0	0
	Lysine Metabolism	20	0.5832	15%	3	2	1	0	0	0	1	0	1
	Methionine, Cysteine, SAM and Taurine Metabolism	22	0.0366	32%	7	5	2	1	1	0	5	4	1
	Phenylalanine Metabolism	7	0.0119	57%	4	0	4	3	0	3	2	0	2
	Polyamine Metabolism	7	0.6728	14%	1	0	1	0	0	0	0	0	0
	Tryptophan Metabolism	23	0.0459	30%	7	1	6	1	1	0	4	1	3
	Tyrosine Metabolism	19	0.5472	16%	3	0	3	2	0	2	1	0	1
	Urea cycle; Arginine and Proline Metabolism	21	0.1924	24%	5	0	5	1	0	1	1	0	1
Carbohydrate	Aminosugar Metabolism	5	0.5503	20%	1	1	0	1	1	0	1	1	0
	Disaccharides and Oligosaccharides	1	1	0%	0	0	0	0	0	0	0	0	0
	Fructose, Mannose and Galactose Metabolism	4	1	0%	0	0	0	0	0	0	0	0	0
	Glycogen Metabolism	2	0.0221	100%	2	2	0	2	2	0	2	2	0
	Glycolysis, Gluconeogenesis, and Pyruvate Metabolism	6	0.0058	67%	4	3	1	1	1	0	3	2	1
	Pentose Metabolism	5	0.5503	20%	1	1	0	0	0	0	1	1	0

Cofactors and Vitamins	Ascorbate and Aldarate Metabolism	6	0.6165	17%	1	1	0	0	0	0	0	0	0
	Hemoglobin and Porphyrin Metabolism	6	1	0%	0	0	0	0	0	0	0	0	0
	Nicotinate and Nicotinamide Metabolism	8	0.1029	38%	3	1	2	1	1	0	2	1	1
	Pantothenate and CoA Metabolism	1	1	0%	0	0	0	0	0	0	0	0	0
	Riboflavin Metabolism	1	1	0%	0	0	0	0	0	0	0	0	0
	Tocopherol Metabolism	6	0.6165	17%	1	0	1	0	0	0	0	0	0
	Vitamin A Metabolism	7	0.0119	57%	4	0	4	0	0	0	0	0	0
	Vitamin B6 Metabolism	2	0.274	50%	1	0	1	0	0	0	1	0	1
Energy	Oxidative Phosphorylation	1	1	0%	0	0	0	0	0	0	0	0	0
	TCA Cycle	9	0.3934	22%	2	2	0	1	1	0	1	1	0
Lipid	Androgenic Steroids	18	1	0%	0	0	0	0	0	0	0	0	0
	Carnitine Metabolism	2	0.274	50%	1	0	1	0	0	0	1	0	1
	Ceramide PEs	1	1	0%	0	0	0	0	0	0	0	0	0
	Corticosteroids	6	1	0%	0	0	0	0	0	0	0	0	0
	Endocannabinoid	8	1	0%	0	0	0	0	0	0	0	0	0
	Fatty Acid Metabolism (Acyl Carnitine, Dicarboxylate)	5	1	0%	0	0	0	0	0	0	0	0	0
	Fatty Acid Metabolism (Acyl Carnitine, Hydroxy)	7	0.6728	14%	1	0	1	0	0	0	0	0	0
	Fatty Acid Metabolism (Acyl Carnitine, Long Chain Saturated)	8	1	0%	0	0	0	0	0	0	0	0	0
	Fatty Acid Metabolism (Acyl Carnitine, Medium Chain)	6	0.0058	67%	4	0	4	0	0	0	4	0	4
	Fatty Acid Metabolism (Acyl Carnitine, Monounsaturated)	10	0.4478	20%	2	0	2	0	0	0	2	0	2
	Fatty Acid Metabolism (Acyl Carnitine, Polyunsaturated)	7	1	0%	0	0	0	0	0	0	0	0	0
	Fatty Acid Metabolism (Acyl Carnitine, Short Chain)	1	1	0%	0	0	0	0	0	0	0	0	0
	Fatty Acid Metabolism (Acyl Choline)	8	1	0%	0	0	0	0	0	0	0	0	0
	Fatty Acid Metabolism (Acyl Glutamine)	1	1	0%	0	0	0	0	0	0	0	0	0
	Fatty Acid Metabolism (Acyl Glycine)	7	1	0%	0	0	0	0	0	0	0	0	0
	Fatty Acid Metabolism (also BCAA Metabolism)	5	1	0%	0	0	0	0	0	0	0	0	0
	Fatty Acid Synthesis	2	0.274	50%	1	1	0	0	0	0	1	1	0
	Fatty Acid, Amide	7	1	0%	0	0	0	0	0	0	0	0	0

	Fatty Acid, Amino	3	1	0%	0	0	0	0	0	0	0	0	0
	Fatty Acid, Branched	4	0.4725	25%	1	0	1	0	0	0	1	0	1
	Fatty Acid, Dicarboxylate	33	0.9635	6%	2	0	2	0	0	0	2	1	1
	Fatty Acid, Dihydroxy	5	0.5503	20%	1	0	1	1	0	1	0	0	0
	Fatty Acid, Monohydroxy	19	1	0%	0	0	0	0	0	0	0	0	0
	Glycerolipid Metabolism	3	0.3813	33%	1	0	1	1	0	1	1	0	1
	Inositol Metabolism	1	1	0%	0	0	0	0	0	0	0	0	0
	Ketone Bodies	2	1	0%	0	0	0	0	0	0	0	0	0
	Long Chain Monounsaturated Fatty Acid	7	1	0%	0	0	0	0	0	0	0	0	0
	Long Chain Polyunsaturated Fatty Acid (n3 and n6)	16	1	0%	0	0	0	0	0	0	0	0	0
	Long Chain Saturated Fatty Acid	6	1	0%	0	0	0	0	0	0	0	0	0
	Lysophospholipid	6	0.6165	17%	1	1	0	0	0	0	1	1	0
	Medium Chain Fatty Acid	10	0.7967	10%	1	1	0	0	0	0	1	1	0
	Mevalonate Metabolism	1	1	0%	0	0	0	0	0	0	0	0	0
	Phosphatidylserine (PS)	2	0.0221	100%	2	2	0	2	2	0	2	2	0
	Phospholipid Metabolism	6	0.0058	67%	4	4	0	2	2	0	3	3	0
	Pregnenolone Steroids	8	1	0%	0	0	0	0	0	0	0	0	0
	Primary Bile Acid Metabolism	11	0.2163	27%	3	0	3	0	0	0	2	0	2
	Progestin Steroids	5	1	0%	0	0	0	0	0	0	0	0	0
	Secondary Bile Acid Metabolism	20	0.0219	35%	7	0	7	2	0	2	6	0	6
	Short Chain Fatty Acid	1	1	0%	0	0	0	0	0	0	0	0	0
	Sphingolipid Synthesis	4	0.0118	75%	3	3	0	3	3	0	2	2	0
	Sphingosines	2	0.0221	100%	2	2	0	2	2	0	2	2	0
	Sterol	8	0.021	50%	4	0	4	2	0	2	3	0	3
Nucleotide	Purine Metabolism, (Hypo)Xanthine/Inosine containing	8	0.1029	38%	3	2	1	1	1	0	2	2	0
	Purine Metabolism, Adenine containing	7	0.2782	29%	2	2	0	1	1	0	2	2	0
	Purine Metabolism, Guanine containing	3	1	0%	0	0	0	0	0	0	0	0	0
	Pyrimidine Metabolism, Cytidine containing	4	1	0%	0	0	0	0	0	0	0	0	0
	Pyrimidine Metabolism, Orotate containing	3	0.3813	33%	1	0	1	1	0	1	1	0	1

	Pyrimidine Metabolism, Thymine containing	2	1	0%	0	0	0	0	0	0	0	0	0
	Pyrimidine Metabolism, Uracil containing	11	0.8264	9%	1	1	0	0	0	0	1	1	0
Partially Characterized Molecules	Partially Characterized Molecules	24	0.8860	8%	2	0	2	0	0	0	1	0	1
Peptide	Acetylated Peptides	3	1	0%	0	0	0	0	0	0	0	0	0
	Dipeptide	9	0.7618	11%	1	1	0	0	0	0	0	0	0
	Dipeptide Derivative	2	1	0%	0	0	0	0	0	0	0	0	0
	Fibrinogen Cleavage Peptide	3	1	0%	0	0	0	0	0	0	0	0	0
	Gamma-glutamyl Amino Acid	16	0.921	6%	1	0	1	1	0	1	0	0	0
	Modified Peptides	1	1	0%	0	0	0	0	0	0	0	0	0
Xenobiotics	Bacterial/Fungal	3	0.3813	33%	1	0	1	0	0	0	0	0	0
	Benzoate Metabolism	25	1	0%	0	0	0	0	0	0	0	0	0
	Chemical	22	0.4142	18%	4	1	3	1	0	1	4	2	2
	Drug - Analgesics, Anesthetics	10	1	0%	0	0	0	0	0	0	0	0	0
	Drug - Antibiotic	3	1	0%	0	0	0	0	0	0	0	0	0
	Drug - Antiinflammatory, Immunosuppressant	1	1	0%	0	0	0	0	0	0	0	0	0
	Drug - Gastrointestinal	2	1	0%	0	0	0	0	0	0	0	0	0
	Drug - Neurological	6	1	0%	0	0	0	0	0	0	0	0	0
	Drug - Psychoactive	6	1	0%	0	0	0	0	0	0	0	0	0
	Drug - Respiratory	3	1	0%	0	0	0	0	0	0	0	0	0
	Drug - Topical Agents	3	1	0%	0	0	0	0	0	0	0	0	0
	Food Component/Plant	60	0.9999	2%	1	0	1	0	0	0	0	0	0
	Xanthine Metabolism	11	1	0%	0	0	0	0	0	0	0	0	0

Supplementary Table S2. Metabolites with differential profiling between SLS and control among all subjects.

Super Pathway	Sub Pathway	Metabolite	KEGG ID[1]	HMDB ID[2]	PUBCHEM ID[3]	SLS Mean	Con-trol Mean	Fold Change	p-value	q-value
Amino Acid	Alanine and Aspartate Metabolism	aspartate	C00049	HMDB0000191	5960	1.4843	0.7738	1.9	9.04E-10	8.35E-08
Amino Acid	Creatine Metabolism	creatinine	C00791	HMDB0000562	588	0.9251	1.1887	0.78	0.0099	0.0381
Amino Acid	Glutamate Metabolism	glutamate	C00025 C00217	HMDB0000148	33032	1.6525	0.8775	1.86	0.0001	0.0012
Amino Acid	Glutamate Metabolism	beta-citrylglutamate	C20775		189741	1.5873	0.5338	2.99	7.24E-10	8.35E-08
Amino Acid	Glutathione Metabolism	cysteinylglycine disulfide*		HMDB0000709	22833544	0.8286	1.1587	0.73	0.0002	0.0016
Amino Acid	Glutathione Metabolism	5-oxoproline	C01879 C02237	HMDB0000267	439685;7405	1.1184	0.9324	1.2	0.0095	0.0371
Amino Acid	Glycine, Serine and Threonine Metabolism	sarcosine	C00213	HMDB0000271	1088	0.808	1.072	0.76	0.0011	0.0072
Amino Acid	Histidine Metabolism	1-methylhistidine	C01152	HMDB0000001	92105	0.7975	1.3245	0.61	0.0022	0.0124
Amino Acid	Histidine Metabolism	3-methylhistidine	C01152	HMDB0000479	64969	1.4774	4.3317	0.36	0.013	0.0454
Amino Acid	Histidine Metabolism	formiminoglutamate	C00439	HMDB0000854	439233	0.7848	1.938	0.41	1.24E-05	0.0002
Amino Acid	Histidine Metabolism	imidazole lactate	C05568	HMDB0002320	793	0.8848	1.416	0.63	0.0043	0.021
Amino Acid	Leucine, Isoleucine and Valine Metabolism	2-methylbutyrylcarnitine (C5)		HMDB0000378	6426901	0.9298	1.4436	0.65	0.0046	0.0211
Amino Acid	Leucine, Isoleucine and Valine Metabolism	tiglylcarnitine (C5:1-DC)		HMDB0002366	22833596	0.727	1.1146	0.66	0.0016	0.0098
Amino Acid	Lysine Metabolism	fructosyllysine		HMDB0034879	9839580	1.3459	0.9152	1.5	0.0134	0.046
Amino Acid	Lysine Metabolism	2-aminoadipate	C00956	HMDB0000510	92136	1.2988	0.871	1.53	0.0025	0.0137
Amino Acid	Lysine Metabolism	pipecolate		HMDB0000070		0.824	1.5496	0.53	6.63E-05	0.0008
Amino Acid	Methionine, Cysteine, SAM and Taurine Metabolism	S-adenosylhomocysteine (SAH)	C00021	HMDB0000939	439155	1.2364	0.7686	1.62	0.0002	0.0017
Amino Acid	Methionine, Cysteine, SAM and Taurine Metabolism	cysteine	C00097	HMDB0000574	5862	0.9103	1.1295	0.81	0.0028	0.0154
Amino Acid	Methionine, Cysteine, SAM and Taurine Metabolism	cysteine s-sulfate	C05824	HMDB0000731	115015	2.6556	0.6574	3.85	0.0013	0.0081
Amino Acid	Methionine, Cysteine, SAM and Taurine Metabolism	cystine	C00491	HMDB0000192	67678	0.8826	1.5085	0.61	0.0056	0.0247
Amino Acid	Methionine, Cysteine, SAM and Taurine Metabolism	hypotaurine	C00519	HMDB0000965	107812	1.8066	0.7744	2.37	0.0002	0.0015
Amino Acid	Methionine, Cysteine, SAM and Taurine Metabolism	taurine	C00245	HMDB0000251	1123	1.5258	0.7966	1.96	6.14E-07	1.53E-05

Amino Acid	Methionine, Cysteine, SAM and Taurine Metabolism	N-acetyltaurine		HMDB0240253	159864	1.2663	0.8356	1.54	0.0013	0.0084
Amino Acid	Phenylalanine Metabolism	phenylalanine	C00079	HMDB0000159	6140	0.9234	1.214	0.76	7.02E-05	0.0008
Amino Acid	Phenylalanine Metabolism	phenylpyruvate	C00166	HMDB0000205	997	0.8035	1.5055	0.52	6.70E-07	1.55E-05
Amino Acid	Phenylalanine Metabolism	phenyllactate (PLA)	C05607	HMDB0000779 HMDB0000563	3848	0.8182	1.3702	0.6	5.42E-06	9.24E-05
Amino Acid	Phenylalanine Metabolism	2-hydroxyphenylacetate	C05852	HMDB0000669	11970	0.9528	1.4715	0.63	0.0148	0.0499
Amino Acid	Polyamine Metabolism	5-methylthioadenosine (MTA)	C00170	HMDB0001173	439176	0.8798	1.1676	0.76	0.0113	0.0421
Amino Acid	Tryptophan Metabolism	tryptophan	C00078	HMDB0000929	6305	0.8634	1.1463	0.75	0.0027	0.0146
Amino Acid	Tryptophan Metabolism	oxindolylalanine			20977764	0.7839	1.3561	0.58	0.0001	0.0014
Amino Acid	Tryptophan Metabolism	kynurenine	C00328	HMDB0000684	161166	0.8012	1.3589	0.61	3.58E-05	0.0005
Amino Acid	Tryptophan Metabolism	kynurenate	C01717	HMDB0000715	3845	0.8091	1.4465	0.57	0.0002	0.0016
Amino Acid	Tryptophan Metabolism	serotonin	C00780	HMDB0000259	5202	3.9609	0.2697	14.68	7.17E-11	4.64E-08
Amino Acid	Tryptophan Metabolism	indolelactate	C02043	HMDB0000671	92904	0.8498	1.2273	0.69	0.0006	0.0044
Amino Acid	Tryptophan Metabolism	indoleacetate	C00954	HMDB0000197	802	0.8855	1.2431	0.71	0.004	0.0203
Amino Acid	Tyrosine Metabolism	tyrosine	C00082	HMDB0000158	6057	0.8897	1.2938	0.67	0.0001	0.0013
Amino Acid	Tyrosine Metabolism	4-hydroxyphenylpyruvate	C01179	HMDB0000707	979	0.8354	1.2543	0.66	0.0006	0.0047
Amino Acid	Tyrosine Metabolism	3-(4-hydroxyphenyl)lactate	C03672	HMDB0000755	9378	0.795	1.3173	0.61	7.35E-06	0.0001
Amino Acid	Urea cycle; Arginine and Proline Metabolism	arginine	C00062	HMDB0000517	6322	0.8412	1.2481	0.68	6.43E-05	0.0008
Amino Acid	Urea cycle; Arginine and Proline Metabolism	2-oxoarginine*	C03771	HMDB0004225	558	0.8672	1.9041	0.46	0.001	0.007
Amino Acid	Urea cycle; Arginine and Proline Metabolism	N-acetylarginine	C02562	HMDB0004620	67427	0.8599	1.1863	0.73	0.0021	0.012
Amino Acid	Urea cycle; Arginine and Proline Metabolism	N,N,N-trimethyl-alanylproline betaine (TMAP)		HMDB0240365		0.9124	1.3388	0.69	0.0005	0.0037

Amino Acid	Urea cycle; Arginine and Proline Metabolism	argininate*		HMDB0003148	160437	0.8797	1.7099	0.51	0.0001	0.0014
Carbohydrate	Aminosugar Metabolism	N-acetylneuraminate	C00270	HMDB0000230	439197	1.3205	0.8617	1.53	1.32E-05	0.0002
Carbohydrate	Glycogen Metabolism	maltotriose	C01835	HMDB0001262	439586	4.3543	0.3364	13.27	2.31E-10	7.47E-08
Carbohydrate	Glycogen Metabolism	maltose	C00208	HMDB0000163	10991489	3.8871	0.463	8.8	5.01E-10	8.35E-08
Carbohydrate	Glycolysis, Gluconeogenesis, and Pyruvate Metabolism	1,5-anhydroglucitol (1,5-AG)	C07326	HMDB0002712	64960	0.8708	1.1722	0.76	0.0074	0.03
Carbohydrate	Glycolysis, Gluconeogenesis, and Pyruvate Metabolism	3-phosphoglycerate	C00597	HMDB0000807	724	2.0847	0.6657	3.22	6.46E-07	1.55E-05
Carbohydrate	Glycolysis, Gluconeogenesis, and Pyruvate Metabolism	pyruvate	C00022	HMDB0000243	1060	1.1295	0.8759	1.27	0.0046	0.0211
Carbohydrate	Glycolysis, Gluconeogenesis, and Pyruvate Metabolism	lactate	C00186	HMDB0000190	612	1.186	0.8871	1.32	0.0032	0.0165
Carbohydrate	Pentose Metabolism	arabonate/xylonate	C00502 C 05411	HMDB0000539	122045;660 2431	1.3651	0.9324	1.44	0.0062	0.0265
Cofactors and Vitamins	Ascorbate and Aldarate Metabolism	gulonate*	C00800	HMDB0003290	152304	1.2412	0.8802	1.41	0.0069	0.0293
Cofactors and Vitamins	Nicotinate and Nicotinamide Metabolism	nicotinamide	C00153	HMDB0001406	936	1.9371	0.6972	2.86	2.58E-08	1.28E-06
Cofactors and Vitamins	Nicotinate and Nicotinamide Metabolism	N1-Methyl-2-pyridone-5-carboxamide	C05842	HMDB0004193	69698	0.8469	1.4076	0.61	0.0014	0.0091
Cofactors and Vitamins	Nicotinate and Nicotinamide Metabolism	N1-Methyl-4-pyridone-3-carboxamide	C05843	HMDB0004194	440810	0.8539	1.5	0.58	0.0019	0.0113
Cofactors and Vitamins	Tocopherol Metabolism	alpha-tocopherol	C02477	HMDB0001893	86472	0.8698	1.1553	0.74	0.0056	0.0247
Cofactors and Vitamins	Vitamin A Metabolism	4-oxo-retinoic acid		HMDB0006285	6437063	0.9592	1.4167	0.67	0.0127	0.0449
Cofactors and Vitamins	Vitamin A Metabolism	carotene diol (1)				1.01	1.3774	0.71	0.0054	0.0244
Cofactors and Vitamins	Vitamin A Metabolism	carotene diol (3)				0.8838	1.4526	0.6	0.0045	0.0211
Cofactors and Vitamins	Vitamin A Metabolism	beta-cryptoxanthin	C08591	HMDB0033844	5281235	0.8142	1.468	0.54	0.0118	0.0427

Cofactors and Vitamins	Vitamin B6 Metabolism	pyridoxal	C00250	HMDB0001545	1050	0.9628	1.1791	0.82	0.0044	0.021
Energy	TCA Cycle	succinate	C00042	HMDB0000254	1110	1.2465	0.923	1.39	0.0002	0.0015
Energy	TCA Cycle	citraconate/glutaconate	C02226 C02214	HMDB0000620 HMDB0000634	643798;5280498	7.687	1.0982	5.95	0.0029	0.0157
Lipid	Carnitine Metabolism	deoxycarnitine	C01181	HMDB0001161	22620	0.8926	1.1402	0.81	0.007	0.0296
Lipid	Fatty Acid Metabolism (Acyl Carnitine, Hydroxy)	3-hydroxydecanoylcarnitine		HMDB0061636	121454166	0.8862	1.4915	0.62	0.0073	0.03
Lipid	Fatty Acid Metabolism (Acyl Carnitine, Medium Chain)	octanoylcarnitine (C8)	C02838	HMDB0000791	11953814;123701	0.8019	2.4033	0.37	0.011	0.0414
Lipid	Fatty Acid Metabolism (Acyl Carnitine, Medium Chain)	cis-3,4-methyleneheptanoylcarnitine				0.799	1.3279	0.63	0.0076	0.0304
Lipid	Fatty Acid Metabolism (Acyl Carnitine, Medium Chain)	decanoylcarnitine (C10)		HMDB0000651	10245190	0.8019	1.9777	0.44	0.0056	0.0247
Lipid	Fatty Acid Metabolism (Acyl Carnitine, Medium Chain)	laurylcarnitine (C12)		HMDB000225	168381	0.9764	1.5437	0.69	0.013	0.0454
Lipid	Fatty Acid Metabolism (Acyl Carnitine, Monounsaturated)	3-decenoylcarnitine				0.6531	1.5317	0.44	4.85E-05	0.0006
Lipid	Fatty Acid Metabolism (Acyl Carnitine, Monounsaturated)	cis-4-decenoylcarnitine (C10:1)		HMDB0013205	57357170	0.8004	1.6496	0.52	0.003	0.0157
Lipid	Fatty Acid Synthesis	malonate	C00383	HMDB0000691	867	1.2266	0.8933	1.36	0.0004	0.0036
Lipid	Fatty Acid, Branched	cis-3,4-methyleneheptanoate				0.8303	1.8365	0.46	0.0002	0.002
Lipid	Fatty Acid, Dicarboxylate	dodecadienoate (12:2)*			25480	0.7953	1.5453	0.52	0.005	0.0229
Lipid	Fatty Acid, Dicarboxylate	3-carboxy-4-methyl-5-pentyl-2-furanpropionate (3-CMPFP)**		HMDB0061643	194501	0.7961	1.309	0.62	0.0017	0.0104
Lipid	Fatty Acid, Dihydroxy	2S,3R-dihydroxybutyrate		HMDB0002453	10964471	0.8529	1.7488	0.48	0.0002	0.0015
Lipid	Glycerolipid Metabolism	glycerol 3-phosphate	C0093	HMDB0000126	754	0.5944	1.433	0.39	9.96E-07	2.08E-05

Lipid	Lysophospholipid	1-linoleoyl-GPA (18:2)*		HMDB0007856		1.8326	0.9602	1.88	0.0009	0.0062
Lipid	Medium Chain Fatty Acid	pelargonate (9:0)	C01601	HMDB0000847	8158	1.4181	0.9537	1.44	0.0136	0.0462
Lipid	Phosphatidylserine (PS)	1-stearoyl-2-oleoyl-GPS (18:0/18:1)		HMDB0010163	9547087	2.4075	0.4016	6.09	1.24E-08	7.32E-07
Lipid	Phosphatidylserine (PS)	1-stearoyl-2-arachidonoyl-GPS (18:0/20:4)		HMDB0012383	24779545	2.5938	0.4599	5.88	1.72E-08	9.26E-07
Lipid	Phospholipid Metabolism	choline phosphate	C00588	HMDB0001565	1014	1.3707	0.8817	1.57	0.0012	0.0081
Lipid	Phospholipid Metabolism	glycerophosphorylcholine (GPC)	C00670	HMDB0000086	71920	1.2875	0.6719	1.9	1.90E-05	0.0003
Lipid	Phospholipid Metabolism	phosphoethanolamine	C00346	HMDB0000224	1015	2.1883	0.7447	3	6.62E-08	2.52E-06
Lipid	Phospholipid Metabolism	glycerophosphoethanolamine	C01233	HMDB0000114	123874	1.4544	0.6938	2.13	4.08E-08	1.88E-06
Lipid	Primary Bile Acid Metabolism	chenodeoxycholate	C02528	HMDB0000518	10133	0.9539	1.6075	0.61	0.0103	0.0391
Lipid	Primary Bile Acid Metabolism	glycochenodeoxycholate	C05466	HMDB0000637	12544	1.0114	4.5185	0.24	0.001	0.0068
Lipid	Primary Bile Acid Metabolism	taurochenodeoxycholate	C05465	HMDB0000951	387316	0.5904	3.0494	0.2	0.0001	0.0012
Lipid	Secondary Bile Acid Metabolism	taurolithocholate 3-sulfate	C03642	HMDB0002580	440071	1.2693	2.0772	0.66	0.0145	0.0491
Lipid	Secondary Bile Acid Metabolism	isoursodeoxycholate	C17662	HMDB0000686	127601	0.7032	2.3023	0.34	0.0005	0.004
Lipid	Secondary Bile Acid Metabolism	glycoursodeoxycholate		HMDB0000708	12310288	1.2404	5.1674	0.25	0.0015	0.0093
Lipid	Secondary Bile Acid Metabolism	glycoursodeoxycholic acid sulfate (1)				0.8349	2.4319	0.36	0.001	0.0067
Lipid	Secondary Bile Acid Metabolism	glycocholenate sulfate*				0.6571	1.9975	0.33	8.63E-07	1.86E-05
Lipid	Secondary Bile Acid Metabolism	taurocholenate sulfate*				0.5922	1.746	0.36	4.82E-08	2.08E-06
Lipid	Secondary Bile Acid Metabolism	taurochenodeoxycholic acid 3-sulfate		HMDB0002486	52931550	0.877	2.1513	0.39	0.002	0.0119
Lipid	Sphingolipid Synthesis	sphinganine	C00836	HMDB0000269	3126	2.417	0.7084	3.51	2.23E-06	4.37E-05
Lipid	Sphingolipid Synthesis	sphinganine-1-phosphate		HMDB0001383	520	1.3847	0.7789	1.78	2.51E-07	6.77E-06
Lipid	Sphingolipid Synthesis	sphingadienine			6449795	2.307	0.8526	2.84	7.96E-05	0.0009
Lipid	Sphingosines	sphingosine	C00319	HMDB0000252	5280335	2.6034	0.5556	4.77	8.15E-08	2.93E-06
Lipid	Sphingosines	sphingosine 1-phosphate	C06124	HMDB0000277	5283560	1.3059	0.841	1.56	7.12E-07	1.59E-05

Lipid	Sterol	cholesterol	C00187	HMDB0000067	6432564;11025495;5997	0.8733	1.1401	0.76	0.0004	0.0036
Lipid	Sterol	7-alpha-hydroxy-3-oxo-4-cholestenoate (7-Hoca)	C17337	HMDB0012458	3081085	0.6451	1.6743	0.4	1.99E-09	1.61E-07
Lipid	Sterol	3beta,7alpha-dihydroxy-5-cholestenoate	C17335	HMDB0012454	3081084	0.7032	1.5325	0.48	4.84E-06	8.69E-05
Lipid	Sterol	3beta-hydroxy-5-cholestenoate	C17333	HMDB0012453	165511	0.5343	2.1681	0.25	7.25E-10	8.35E-08
Nucleotide	Purine Metabolism, (Hypo)Xanthine/Inosine containing	hypoxanthine	C00262	HMDB0000157	790;135398638	1.2128	0.8315	1.42	0.0036	0.0187
Nucleotide	Purine Metabolism, (Hypo)Xanthine/Inosine containing	xanthine	C00385	HMDB0000292	1188	1.4204	0.7427	1.94	3.32E-09	2.39E-07
Nucleotide	Purine Metabolism, (Hypo)Xanthine/Inosine containing	urate	C00366	HMDB0000289	1175	0.9406	1.1725	0.81	0.0124	0.0439
Nucleotide	Purine Metabolism, Adenine containing	adenosine 5'-monophosphate (AMP)	C00020	HMDB0000045	6083	2.0149	0.5184	4.02	1.67E-07	4.92E-06
Nucleotide	Purine Metabolism, Adenine containing	adenine	C00147	HMDB0000034	190	1.9322	0.8615	2.1	0.0009	0.0062
Nucleotide	Pyrimidine Metabolism, Orotate containing	dihydroorotate	C00337	HMDB03349	648	0.5993	1.1933	0.48	7.33E-06	0.0001
Nucleotide	Pyrimidine Metabolism, Uracil containing	uracil	C00106	HMDB0000300	1174	1.3497	0.7391	1.82	9.16E-06	0.0001
Partially Characterized Molecules	Partially Characterized Molecules	glycine conjugate of C10H14O2 (1)*				0.8782	1.5933	0.56	0.0017	0.0102
Partially Characterized Molecules	Partially Characterized Molecules	branched-chain, straight-chain, or cyclopropyl 10:1 fatty acid (2)*				0.742	1.3714	0.55	0.0072	0.0297
Peptide	Dipeptide	leucylglycine		HMDB0028929	79070	1.499	0.9072	1.67	0.0055	0.0247
Peptide	Gamma-glutamyl Amino Acid	gamma-glutamyltyrosine		HMDB0011741	94340	0.8765	1.3029	0.66	0.0008	0.0061
Xenobiotics	Bacterial/Fungal	N-methylpipecolate			11286529	1.097	1.731	0.65	0.0103	0.0391
Xenobiotics	Chemical	4-methylbenzenesulfonate	C06677	HMDB0059933	85570	1.1723	0.8294	1.38	0.0002	0.002
Xenobiotics	Chemical	thioproline		HMDB0062164	93176	0.7956	1.1752	0.7	1.06E-05	0.0002

Xenobiotics	Chemical	3,5-dichloro-2,6-dihydroxybenzoic acid			24721632	0.8517	1.4129	0.6	0.0026	0.0142
Xenobiotics	Chemical	3-bromo-5-chloro-2,6-dihydroxybenzoic acid *			134581543	0.6726	1.5768	0.42	1.46E-06	2.96E-05
Xenobiotics	Food Component/Plant	3-formylindole	C08493	HMDB29737	10256	0.9377	1.2702	0.73	0.0044	0.021

[1] KEGG: Kyoto Encyclopedia of Genes and Genomes; https://www.genome.jp/kegg
[2] Human Metabolome Database; https://hmdb.ca
[3] PubChem; https://pubchem.ncbi.nlm.nih.gov

Supplementary Table S3. Metabolites with differential profiling between SLS and control among females.

Super Pathway	Sub Pathway	Metabolite	KEGG ID[1]	HMDB ID[2]	PUBCHEM ID[3]	Female SLS Mean	Female Control Mean	Fold Change	p-Value	q-Value
Amino Acid	Alanine and Aspartate Metabolism	aspartate	C00049	HMDB0000191	5960	1.535	0.762	2.01	6.13E-08	1.34E-05
Amino Acid	Glutamate Metabolism	glutamate	C00025 C00217	HMDB0000148	33032	1.7526	0.8791	1.99	0.0006	0.0067
Amino Acid	Glutamate Metabolism	beta-citrylglutamate	C20775		189741	1.5292	0.5094	3	3.07E-07	2.75E-05
Amino Acid	Glutathione Metabolism	cysteinylglycine disulfide*		HMDB0000709	22833544	0.7156	1.0717	0.67	0.0007	0.0075
Amino Acid	Glycine, Serine and Threonine Metabolism	sarcosine	C00213	HMDB0000271	1088	0.7945	1.0851	0.73	0.0065	0.038
Amino Acid	Glycine, Serine and Threonine Metabolism	N-acetylserine		HMDB0002931	65249	1.1516	0.8434	1.37	0.0064	0.0378
Amino Acid	Histidine Metabolism	formiminoglutamate	C00439	HMDB0000854	439233	0.6341	1.4941	0.42	0.0017	0.0143
Amino Acid	Lysine Metabolism	pipecolate		HMDB0000070		0.8828	1.7288	0.51	0.0014	0.0122
Amino Acid	Methionine, Cysteine, SAM and Taurine Metabolism	S-adenosylhomocysteine (SAH)	C00021	HMDB0000939	439155	1.1569	0.6995	1.65	0.0013	0.012
Amino Acid	Methionine, Cysteine, SAM and Taurine Metabolism	cysteine s-sulfate	C05824	HMDB0000731	115015	2.9731	0.544	5.47	0.0005	0.0058

Amino Acid	Methionine, Cysteine, SAM and Taurine Metabolism	cystine	C00491	HMDB0000192	67678	0.7228	1.5371	0.47	0.0023	0.0176
Amino Acid	Methionine, Cysteine, SAM and Taurine Metabolism	hypotaurine	C00519	HMDB0000965	107812	1.6271	0.7122	2.28	0.0029	0.021
Amino Acid	Methionine, Cysteine, SAM and Taurine Metabolism	taurine	C00245	HMDB0000251	1123	1.4343	0.8699	1.65	0.0019	0.0161
Amino Acid	Phenylalanine Metabolism	phenylpyruvate	C00166	HMDB0000205	997	0.8377	1.4154	0.59	0.0008	0.0079
Amino Acid	Phenylalanine Metabolism	phenyllactate (PLA)	C05607	HMDB0000779 HMDB0000563	3848	0.7452	1.1763	0.63	0.0007	0.0075
Amino Acid	Tryptophan Metabolism	oxindolylalanine			20977764	0.8253	1.5425	0.54	0.0023	0.0176
Amino Acid	Tryptophan Metabolism	kynurenine	C00328	HMDB0000684	161166	0.7009	1.3082	0.54	9.76E-05	0.0019
Amino Acid	Tryptophan Metabolism	kynurenate	C01717	HMDB0000715	3845	0.6637	1.1326	0.59	0.0055	0.0348
Amino Acid	Tryptophan Metabolism	serotonin	C00780	HMDB0000259	5202	3.6352	0.2185	16.64	1.21E-08	7.90E-06
Amino Acid	Tyrosine Metabolism	3-(4-hydroxyphenyl)lactate	C03672	HMDB0000755	9378	0.7286	1.103	0.66	0.0016	0.014
Amino Acid	Urea cycle; Arginine and Proline Metabolism	arginine	C00062	HMDB0000517	6322	0.8252	1.2422	0.66	0.0014	0.0122
Carbohydrate	Aminosugar Metabolism	N-acetylneuraminate	C00270	HMDB0000230	439197	1.3189	0.8423	1.57	0.0003	0.0044
Carbohydrate	Glycogen Metabolism	maltotriose	C01835	HMDB0001262	439586	3.5655	0.2772	12.86	2.21E-07	2.75E-05
Carbohydrate	Glycogen Metabolism	maltose	C00208	HMDB0000163	10991489	2.9058	0.405	7.17	5.96E-07	3.91E-05
Carbohydrate	Glycolysis, Gluconeogenesis, and Pyruvate Metabolism	1,5-anhydroglucitol (1,5-AG)	C07326	HMDB0002712	64960	0.7922	1.196	0.66	0.008	0.0438
Carbohydrate	Glycolysis, Gluconeogenesis, and Pyruvate Metabolism	3-phosphoglycerate	C00597	HMDB0000807	724	1.8305	0.6563	2.79	0.0002	0.0033
Carbohydrate	Glycolysis, Gluconeogenesis, and Pyruvate Metabolism	pyruvate	C00022	HMDB0000243	1060	1.1932	0.875	1.36	0.0079	0.0438
Carbohydrate	Pentose Metabolism	arabonate/xylonate	C00502 C05411	HMDB0000539	122045;6602431	1.4072	0.8505	1.65	0.0046	0.0305
Cofactors and Vitamins	Nicotinate and Nicotinamide Metabolism	nicotinamide	C00153	HMDB0001406	936	1.7176	0.712	2.41	9.05E-05	0.0018
Cofactors and Vitamins	Nicotinate and Nicotinamide Metabolism	N1-Methyl-4-pyridone-3-carboxamide	C05843	HMDB0004194	440810	0.7164	1.3138	0.55	0.0066	0.0382
Cofactors and Vitamins	Vitamin B6 Metabolism	pyridoxal	C00250	HMDB0001545	1050	0.9206	1.1677	0.79	0.0077	0.0432

Energy	TCA Cycle	citraconate/glutaconate	C02226 C02214	HMDB0000620 HMDB0000634	643798;5280498	11.3406	0.7424	15.28	0.0012	0.0119
Lipid	Carnitine Metabolism	deoxycarnitine	C01181	HMDB0001161	22620	0.7452	1.059	0.7	0.0075	0.0427
Lipid	Fatty Acid Metabolism (Acyl Carnitine, Medium Chain)	octanoylcarnitine (C8)	C02838	HMDB0000791	11953814;123701	0.6004	3.1366	0.19	0.0089	0.0464
Lipid	Fatty Acid Metabolism (Acyl Carnitine, Medium Chain)	cis-3,4-methyleneheptanoylcarnitine				0.6662	1.4556	0.46	0.0004	0.0056
Lipid	Fatty Acid Metabolism (Acyl Carnitine, Medium Chain)	decanoylcarnitine (C10)		HMDB0000651	10245190	0.6072	2.3174	0.26	0.0048	0.0318
Lipid	Fatty Acid Metabolism (Acyl Carnitine, Medium Chain)	laurylcarnitine (C12)		HMDB000225	168381	0.6062	1.566	0.39	0.0054	0.0348
Lipid	Fatty Acid Metabolism (Acyl Carnitine, Monounsaturated)	3-decenoylcarnitine				0.5125	1.3059	0.39	0.0005	0.0058
Lipid	Fatty Acid Metabolism (Acyl Carnitine, Monounsaturated)	cis-4-decenoylcarnitine (C10:1)		HMDB0013205	57357170	0.6351	1.7909	0.35	0.0012	0.0119
Lipid	Fatty Acid Synthesis	malonate	C00383	HMDB0000691	867	1.2626	0.8597	1.47	0.0007	0.0075
Lipid	Fatty Acid, Branched	cis-3,4-methyleneheptanoate				0.7647	1.974	0.39	0.0001	0.0025
Lipid	Fatty Acid, Dicarboxylate	maleate	C01384	HMDB0000176	444266	2.1353	0.8079	2.64	0.0006	0.0071
Lipid	Fatty Acid, Dicarboxylate	3-carboxy-4-methyl-5-pentyl-2-furanpropionate (3-CMPFP)**		HMDB0061643	194501	0.7147	1.3041	0.55	0.0009	0.0096
Lipid	Glycerolipid Metabolism	glycerol 3-phosphate	C0093	HMDB0000126	754	0.6998	1.3574	0.52	0.0022	0.0173
Lipid	Lysophospholipid	1-linoleoyl-GPA (18:2)*		HMDB0007856		2.0038	1.0154	1.97	0.0044	0.03
Lipid	Medium Chain Fatty Acid	pelargonate (9:0)	C01601	HMDB0000847	8158	1.5537	0.8683	1.79	0.0053	0.0348
Lipid	Phosphatidylserine (PS)	1-stearoyl-2-oleoyl-GPS (18:0/18:1)		HMDB0010163	9547087	2.2537	0.3987	5.65	1.49E-05	0.0006
Lipid	Phosphatidylserine (PS)	1-stearoyl-2-arachidonoyl-GPS (18:0/20:4)		HMDB0012383	24779545	2.1269	0.4525	4.7	2.58E-05	0.0008
Lipid	Phospholipid Metabolism	glycerophosphorylcholine (GPC)	C00670	HMDB0000086	71920	1.3001	0.6326	2.06	0.0003	0.0039
Lipid	Phospholipid Metabolism	phosphoethanolamine	C00346	HMDB0000224	1015	2.0606	0.7841	2.63	7.07E-05	0.0017
Lipid	Phospholipid Metabolism	glycerophosphoethanolamine	C01233	HMDB0000114	123874	1.3901	0.713	1.95	4.93E-05	0.0013

Lipid	Primary Bile Acid Metabolism	glycochenodeoxycholate	C05466	HMDB0000637	12544	0.8511	5.8661	0.15	0.0026	0.0194
Lipid	Primary Bile Acid Metabolism	taurochenodeoxycholate	C05465	HMDB0000951	387316	0.6042	4.0871	0.15	0.0004	0.0056
Lipid	Secondary Bile Acid Metabolism	deoxycholic acid glucuronide				0.6728	1.8125	0.37	0.0078	0.0438
Lipid	Secondary Bile Acid Metabolism	isoursodeoxycholate	C17662	HMDB0000686	127601	0.3832	2.1066	0.18	7.73E-05	0.0017
Lipid	Secondary Bile Acid Metabolism	glycoursodeoxycholate		HMDB0000708	12310288	1.1702	6.0858	0.19	0.0012	0.0119
Lipid	Secondary Bile Acid Metabolism	glycoursodeoxycholic acid sulfate (1)				0.6473	2.2132	0.29	0.0028	0.0201
Lipid	Secondary Bile Acid Metabolism	glycocholenate sulfate*				0.5977	1.8465	0.32	0.0001	0.0021
Lipid	Secondary Bile Acid Metabolism	taurocholenate sulfate*				0.4462	1.847	0.24	5.56E-07	3.91E-05
Lipid	Sphingolipid Synthesis	sphinganine	C00836	HMDB0000269	3126	2.1048	0.6908	3.05	0.0007	0.0079
Lipid	Sphingolipid Synthesis	sphinganine-1-phosphate		HMDB0001383	520	1.3804	0.7771	1.78	4.73E-05	0.0013
Lipid	Sphingosines	sphingosine	C00319	HMDB0000252	5280335	2.3523	0.5244	4.49	3.06E-05	0.0009
Lipid	Sphingosines	sphingosine 1-phosphate	C06124	HMDB0000277	5283560	1.2737	0.8136	1.57	7.75E-05	0.0017
Lipid	Sterol	7-alpha-hydroxy-3-oxo-4-cholestenoate (7-Hoca)	C17337	HMDB0012458	3081085	0.5313	1.4417	0.37	3.19E-07	2.75E-05
Lipid	Sterol	3beta,7alpha-dihydroxy-5-cholestenoate	C17335	HMDB0012454	3081084	0.5725	1.5555	0.37	9.40E-06	0.0004
Lipid	Sterol	3beta-hydroxy-5-cholestenoate	C17333	HMDB0012453	165511	0.4736	2.1351	0.22	6.07E-08	1.34E-05
Nucleotide	Purine Metabolism, (Hypo)Xanthine/Inosine containing	hypoxanthine	C00262	HMDB0000157	790;135398638	1.3551	0.8395	1.61	0.0027	0.0199
Nucleotide	Purine Metabolism, (Hypo)Xanthine/Inosine containing	xanthine	C00385	HMDB0000292	1188	1.3161	0.7278	1.81	6.17E-06	0.0003
Nucleotide	Purine Metabolism, Adenine containing	adenosine 5'-monophosphate (AMP)	C00020	HMDB0000045	6083	1.7659	0.5394	3.27	8.64E-05	0.0018
Nucleotide	Purine Metabolism, Adenine containing	adenine	C00147	HMDB0000034	190	2.3775	0.8053	2.95	0.0003	0.004
Nucleotide	Pyrimidine Metabolism, Orotate containing	dihydroorotate	C00337	HMDB03349	648	0.6941	1.1519	0.6	0.0089	0.0464
Nucleotide	Pyrimidine Metabolism, Uracil containing	uracil	C00106	HMDB0000300	1174	1.3275	0.6813	1.95	6.05E-05	0.0015
Partially Characterized Molecules	Partially Characterized Molecules	glycine conjugate of C10H14O2 (1)*				0.8401	1.672	0.5	0.002	0.0163

Xenobiotics	Chemical	sulfate*	C00059	HMDB01448	5152822	1.2196	0.9155	1.33	0.0054	0.0348
Xenobiotics	Chemical	4-methylbenzenesulfonate	C06677	HMDB0059933	85570	1.2643	0.8026	1.58	0.0001	0.0021
Xenobiotics	Chemical	thioproline		HMDB0062164	93176	0.7233	1.2367	0.58	3.39E-06	0.0002
Xenobiotics	Chemical	3-bromo-5-chloro-2,6-dihydroxybenzoic acid*			134581543	0.6709	1.3467	0.5	0.0017	0.0147

[1] KEGG: Kyoto Encyclopedia of Genes and Genomes; https://www.genome.jp/kegg

[2] Human Metabolome Database; https://hmdb.ca

[3] PubChem; https://pubchem.ncbi.nlm.nih.gov

Supplementary Table S4. Metabolites with differential profiling between SLS and control among males.

Super Pathway	Sub Pathway	Metabolite	KEGG ID[1]	HMDB ID[2]	PUB-CHEM ID[3]	Male SLS (mean)	Male Control (mean)	Fold Change	p-value	q-value
Amino Acid	Alanine and Aspartate Metabolism	aspartate	C00049	HMDB0000191	5960	1.4082	0.7883	1.79	1.55E-05	0.0006
Amino Acid	Glutamate Metabolism	beta-citrylglutamate	C20775		189741	1.6745	0.5636	2.97	2.82E-06	0.0003
Amino Acid	Histidine Metabolism	formiminoglutamate	C00439	HMDB0000854	439233	1.0108	2.4806	0.41	0.0006	0.0116
Amino Acid	Methionine, Cysteine, SAM and Taurine Metabolism	taurine	C00245	HMDB0000251	1123	1.663	0.7069	2.35	1.15E-05	0.0005
Amino Acid	Phenylalanine Metabolism	phenylalanine	C00079	HMDB0000159	6140	0.9093	1.264	0.72	0.001	0.0178
Amino Acid	Phenylalanine Metabolism	phenylpyruvate	C00166	HMDB0000205	997	0.7523	1.6156	0.47	3.11E-05	0.0012
Amino Acid	Phenylalanine Metabolism	phenyllactate (PLA)	C05607	HMDB0000779 HMDB0000563	3848	0.9275	1.6073	0.58	0.0005	0.0094
Amino Acid	Tryptophan Metabolism	serotonin	C00780	HMDB0000259	5202	4.4494	0.3324	13.39	1.74E-06	0.0002
Amino Acid	Tyrosine Metabolism	tyrosine	C00082	HMDB0000158	6057	0.7942	1.3463	0.59	0.0007	0.0131
Amino Acid	Tyrosine Metabolism	3-(4-hydroxyphenyl)lactate	C03672	HMDB0000755	9378	0.8946	1.5792	0.57	0.0003	0.0076
Amino Acid	Urea cycle; Arginine and Proline Metabolism	argininate*		HMDB0003148	160437	0.966	2.3137	0.42	0.0005	0.0094
Carbohydrate	Aminosugar Metabolism	N-acetylneuraminate	C00270	HMDB0000230	439197	1.323	0.8854	1.49	0.0027	0.0434
Carbohydrate	Glycogen Metabolism	maltotriose	C01835	HMDB0001262	439586	5.5373	0.4087	13.55	7.40E-07	0.0002

Carbohydrate	Glycogen Metabolism	maltose	C00208	HMDB0000163	10991489	5.3591	0.534	10.04	9.45E-07	0.0002
Carbohydrate	Glycolysis, Gluconeogenesis, and Pyruvate Metabolism	3-phosphoglycerate	C00597	HMDB0000807	724	2.466	0.6771	3.64	9.15E-05	0.0027
Cofactors and Vitamins	Nicotinate and Nicotinamide Metabolism	nicotinamide	C00153	HMDB0001406	936	2.2664	0.6791	3.34	2.79E-06	0.0003
Energy	TCA Cycle	succinate	C00042	HMDB0000254	1110	1.4701	0.9659	1.52	0.0014	0.0243
Lipid	Fatty Acid, Dihydroxy	2S,3R-dihydroxybutyrate		HMDB0002453	10964471	0.984	2.3552	0.42	0.0004	0.009
Lipid	Glycerolipid Metabolism	glycerol 3-phosphate	C0093	HMDB0000126	754	0.4363	1.5252	0.29	1.92E-05	0.0008
Lipid	Phosphatidylserine (PS)	1-stearoyl-2-oleoyl-GPS (18:0/18:1)		HMDB0010163	9547087	2.6382	0.4052	6.51	5.03E-06	0.0003
Lipid	Phosphatidylserine (PS)	1-stearoyl-2-arachidonoyl-GPS (18:0/20:4)		HMDB0012383	24779545	3.2943	0.4688	7.03	4.85E-06	0.0003
Lipid	Phospholipid Metabolism	phosphoethanolamine	C00346	HMDB0000224	1015	2.3799	0.6966	3.42	1.23E-05	0.0005
Lipid	Phospholipid Metabolism	glycerophosphoethanolamine	C01233	HMDB0000114	123874	1.5508	0.6704	2.31	8.80E-06	0.0005
Lipid	Secondary Bile Acid Metabolism	glycocholenate sulfate*				0.7463	2.182	0.34	0.0002	0.0053
Lipid	Secondary Bile Acid Metabolism	taurocholenate sulfate*				0.811	1.6225	0.5	0.0005	0.0094
Lipid	Sphingolipid Synthesis	sphinganine	C00836	HMDB0000269	3126	2.8852	0.7299	3.95	0.0001	0.004
Lipid	Sphingolipid Synthesis	sphinganine-1-phosphate		HMDB0001383	520	1.3911	0.7811	1.78	9.93E-05	0.0028
Lipid	Sphingolipid Synthesis	sphingadienine			6449795	2.89	0.7954	3.63	0.0004	0.009
Lipid	Sphingosines	sphingosine	C00319	HMDB0000252	5280335	2.9801	0.5937	5.02	3.33E-05	0.0012
Lipid	Sphingosines	sphingosine 1-phosphate	C06124	HMDB0000277	5283560	1.3543	0.8744	1.55	0.0002	0.0058
Lipid	Sterol	7-alpha-hydroxy-3-oxo-4-cholestenoate (7-Hoca)	C17337	HMDB0012458	3081085	0.8157	1.9587	0.42	1.11E-05	0.0005
Lipid	Sterol	3beta-hydroxy-5-cholestenoate	C17333	HMDB0012453	165511	0.6254	2.2086	0.28	1.15E-05	0.0005
Nucleotide	Purine Metabolism, (Hypo)Xanthine/Inosine containing	xanthine	C00385	HMDB0000292	1188	1.5767	0.761	2.07	1.77E-06	0.0002
Nucleotide	Purine Metabolism, Adenine containing	adenosine 5'-monophosphate (AMP)	C00020	HMDB0000045	6083	2.3884	0.4927	4.85	3.49E-05	0.0012
Nucleotide	Pyrimidine Metabolism, Orotate containing	dihydroorotate	C00337	HMDB03349	648	0.4571	1.2439	0.37	6.38E-05	0.002
Peptide	Gamma-glutamyl Amino Acid	gamma-glutamyltyrosine		HMDB0011741	94340	0.8283	1.5179	0.55	0.002	0.0347
Xenobiotics	Chemical	3-bromo-5-chloro-2,6-dihydroxybenzoic acid*			134581543	0.6752	1.858	0.36	3.96E-05	0.0013

[1] KEGG: Kyoto Encyclopedia of Genes and Genomes; https://www.genome.jp/kegg

[2] Human Metabolome Database; https://hmdb.ca

[3] PubChem; https://pubchem.ncbi.nlm.nih.gov

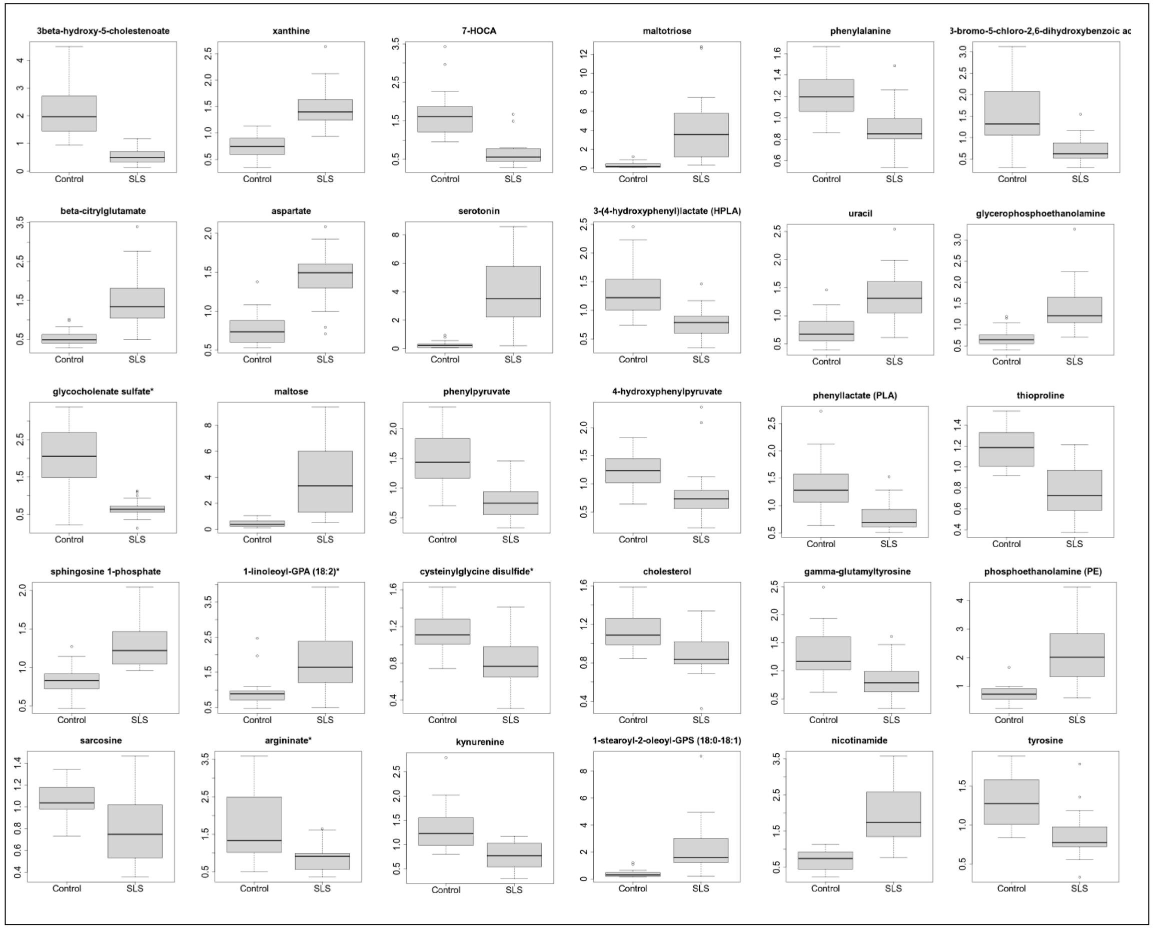

Figure S1. Boxplot comparisons of SLS and controls for metabolites in Random forest analysis (Figure 1). The Y-axis in all box plots corresponds to Scaled Intensity of normalized raw data (see Procedures, Metabolomic Analysis), not SLS/Control ratios**.**

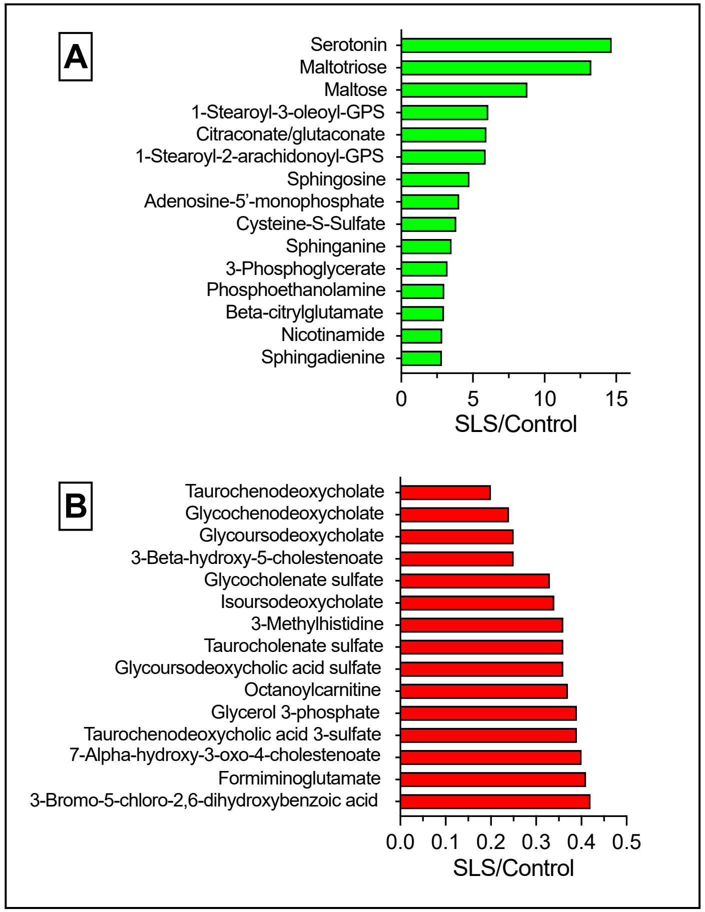

Figure S2. Top 15 metabolites that were increased or decreased in SLS. (**A**). Increased metabolites. (**B**). Decreased metabolites. X-axis represents mean SLS/Control ratio.
